# Supplementary material for: SPACE FOR COPD delivered as a maintenance programme on pulmonary rehabilitation discharge: protocol of a randomised controlled trial evaluating the long-term effects on exercise tolerance and mental well-being
Source: BMJ Open. 2022 Apr 25;12(4):e055513. doi: 10.1136/bmjopen-2021-055513 (PMC9039383; doi:10.1136/bmjopen-2021-055513)
Supplement: Supplementary data [file bmjopen-2021-055513supp001.pdf]

NIHR | Leicester Biomedical Research Centre

Leicester Biomedical Research Centre
Respiratory
Glenfield Hospital
Groby Road
Leicester
LE3 9QP
Tel: 0116 258 3370
Fax: 0116 258 3494
Twitter:@leicesterbrc
Email: NIHRleicesterbrc@uhl-tr.nhs.uk
www.leicesterbrc.nihr.ac.uk

CONSENT FORM FOR PARTICIPANTS

(Version 0.5 on 09/04/2021)

Trial Title: SPACE FOR COPD© delivered as a maintenance programme on Pulmonary Rehabilitation discharge: a randomised controlled trial evaluating the long-term effects on exercise tolerance and mental wellbeing

**Site:**	
**Participant Trial ID number:**	

Please **initial** box

1. I confirm that I have read and understand the information sheet **version 0.5 09/04/2021**, for the above trial, and have had the opportunity to ask questions. ☐
2. I understand that my participation is voluntary and that I am free to withdraw at any time without giving a reason, and without my medical care or legal rights being affected. I understand that any data collected up until my withdrawal will still be used in the research trial. ☐
3. I agree to undergo the tests and investigations described in the Participant Information Sheet. The nature of the tests and investigations and any possible risks have been explained. ☐
4. I understand that relevant sections of my medical notes and data collected during the trial may be looked at by individuals from the trial team, the Sponsor, the funder, regulatory authorities or the NHS Trust, where it is relevant to my taking part in this research. I give permission for these individuals to have access to my records. ☐
5. I give permission for the clinical data collected during my initial and discharge assessments from my most recent Pulmonary Rehabilitation attendance to be retrieved by the trial team. ☐
6. I give permission to the trial team to retrieve data about the healthcare services I have utilised over the 12 months of the trial. ☐
7. I understand that some of my data will be shared with Insignia Health and entered onto a secure database to help improve patient outcome measures, if it is anonymised. ☐

➔➔➔

NIHR | Leicester Biomedical Research Centre

Please **initial** box

8. I understand that my personal details and trial data will be stored on password protected computer systems and paper copies will be kept in a secure office environment.

9. I understand that the information collected about me will be used to support other research in the future, and may be shared anonymously with other researchers.

10. I agree to take part in the above trial. Yes No

11. I agree to take part in the interview at the end of the SPACE maintenance programme which will be audio recorded and transcribed for research purposes. I also agree that anonymised direct quotes can be used for research purposes. Yes No

Name of participant (printed)	Date	Signature
Name of person taking consent (printed)	Date	Signature

Consent form completed: by telephone/ face to face (delete as appropriate)

Original in the TMF Copy to the participant and Copy in medical notes

Alqahtani KA, *et al. BMJ Open* 2022; 12:e055513. doi: 10.1136/bmjopen-2021-055513
