# Supplementary material for: SPACE FOR COPD delivered as a maintenance programme on pulmonary rehabilitation discharge: protocol of a randomised controlled trial evaluating the long-term effects on exercise tolerance and mental well-being
Source: BMJ Open. 2022 Apr 25;12(4):e055513. doi: 10.1136/bmjopen-2021-055513 (PMC9039383; doi:10.1136/bmjopen-2021-055513)
Supplement: Author's manuscript [file bmjopen-2021-055513.draft_revisions.pdf]

BMJ Open is committed to open peer review. As part of this commitment we make the peer review history of every article we publish publicly available.

When an article is published we post the peer reviewers' comments and the authors' responses online. We also post the versions of the paper that were used during peer review. These are the versions that the peer review comments apply to.

The versions of the paper that follow are the versions that were submitted during the peer review process. They are not the versions of record or the final published versions. They should not be cited or distributed as the published version of this manuscript.

BMJ Open is an open access journal and the full, final, typeset and author-corrected version of record of the manuscript is available on our site with no access controls, subscription charges or pay-per-view fees (http://bmjopen.bmj.com).

If you have any questions on BMJ Open's open peer review process please email info.bmjopen@bmj.com

BMJ Open

SPACE FOR COPD© delivered as a maintenance programme on Pulmonary Rehabilitation discharge: protocol of a randomised controlled trial evaluating the long-term effects on exercise tolerance and mental wellbeing.

Journal:	*BMJ Open*
Manuscript ID	bmjopen-2021-055513
Article Type:	Protocol
Date Submitted by the Author:	02-Aug-2021

Complete List of Authors:	Alqahtani, Khaled A.; Glenfield Hospital Respiratory Medicine Department, Centre of Exercise and Rehabilitation Science, Leicester Biomedical Research Centre- Respiratory; University of Leicester, Respiratory Sciences<br>Gerlis, Charlotte; Glenfield Hospital Respiratory Medicine Department, Centre of Exercise and Rehabilitation Science, Leicester Biomedical Research Centre- Respiratory<br>Nolan, Claire; Royal Brompton and Harefield NHS Foundation Trust, Department of Respiratory Medicine<br>Gardiner, Nikki; Glenfield Hospital Respiratory Medicine Department, Centre of Exercise and Rehabilitation Science, Leicester Biomedical Research Centre- Respiratory<br>Szczepura, Ala; Coventry University, Faculty of Health and Life Sciences<br>Man, William; Royal Brompton and Harefield NHS Foundation Trust, Respiratory Medicine; Imperial College London Faculty of Medicine<br>Singh, Sally; Glenfield Hospital Respiratory Medicine Department, Centre for Exercise and Rehabilitation Science, Leicester Biomedical Research Centre-Respiratory; University of Leicester, Respiratory Sciences<br>Houchen-Wolloff, Linzy; Glenfield Hospital Respiratory Medicine Department, Centre of Exercise and Rehabilitation Science, Leicester Biomedical Research Centre- Respiratory; University of Leicester, Respiratory Sciences

Keywords:	Protocols & guidelines < HEALTH SERVICES ADMINISTRATION & MANAGEMENT, QUALITATIVE RESEARCH, REHABILITATION MEDICINE, RESPIRATORY MEDICINE (see Thoracic Medicine), Chronic airways disease < THORACIC MEDICINE

SCHOLARONE™
Manuscripts

TITLE PAGE

Authors: Khaled Alqahtani[1,2], Charlotte Gerlis[1], Claire Nolan[3], Nikki Gardiner[1], Ala Szczepura[4], William Man[3,5], Sally J Singh[1,2], Linzy Houchen-Wolloff[1,2].

Corresponding author details: Linzy Houchen-Wolloff PhD. Address: Glenfield Hospital Respiratory Medicine Department, Centre of Exercise and Rehabilitation Science, Leicester Biomedical Research Centre- Respiratory, Leicester, UK. Telephone: 0044 0116 250 2759. Email: Linzy.Houchen@uhl-tr.nhs.uk Fax: n/a.

Co-author details:

1 Glenfield Hospital Respiratory Medicine Department, Centre of Exercise and Rehabilitation Science, Leicester Biomedical Research Centre- Respiratory, Leicester, UK.

2 University of Leicester, Respiratory Sciences, Leicester, UK.

3 Royal Brompton and Harefield NHS Foundation Trust, Respiratory Medicine, London, UK.

4 Coventry University, Faculty of Health and Life Sciences, Coventry, West Midlands, UK.

5 Imperial College London Faculty of Medicine, London, UK.

Key words: Protocols & guidelines, Rehabilitation medicine, Respiratory medicine, Chronic airways disease, Qualitative research.

Word count: 4854

For peer review only

TITLE

SPACE FOR COPD© delivered as a maintenance programme on Pulmonary Rehabilitation discharge: protocol of a randomised controlled trial evaluating the long-term effects on exercise tolerance and mental wellbeing.

ABSTRACT

Introduction The benefits achieved during pulmonary rehabilitation (PR) are known to be sustained for 6-12 months after the initial programme. Several maintenance trials have been conducted but were heterogeneous in terms of duration, frequency and labour cost. There is no consensus on one best strategy. SPACE FOR COPD© (Self-management Programme of Activity, Coping, and Education) is a home-based self-management programme which has been shown previously to be effective in primary and secondary care settings and is to be tested here as a maintenance programme. The aim is to evaluate the efficacy of the SPACE FOR COPD© programme (manual and group sessions), on exercise tolerance and mental wellbeing, compared to usual care following PR in patients with COPD.

Methods and analysis A prospective, multicentre, single-blinded randomised controlled trial requiring 116 participants with a clinical diagnosis of COPD who have finished PR within four weeks will be randomised 1:1 to either a usual care group or a SPACE FOR COPD© programme group. The intervention comprises a home-based manual and four, two-hour group sessions adopting motivational interviewing techniques over 12-months. The primary outcome is endurance capacity measured by the Endurance Shuttle Walking Test at 12 months. Secondary outcomes are; maximal exercise capacity, health-related quality of life, mood, patient activation, physical activity, lung function, and health care costs. The measures will be taken at baseline, 6- and 12-months. Patient interviews and staff focus groups will be conducted to explore barriers, facilitators, and views about the intervention at the end of the study. A framework analysis will be used for the interpretation of qualitative data.

Ethics and dissemination The trial was granted ethical approval from Health Research Authority and Health and Care Research Wales (HCRW19/EM/0267 on 14 Oct 2019). Results will be made available to all stakeholders through a dissemination event, conferences, and peer-reviewed publications.

Trial registration ISRCTN30110012.

For peer review only - http://bmjopen.bmj.com/site/about/guidelines.xhtml

STRENGTHS AND LIMITATIONS OF THIS STUDY

- This is a novel maintenance trial that uses an evidence-based home-based manual integrated with motivational interviewing skills to promote long-term self-management and behavioural change.
- Patients with COPD who are registered in our patient and public involvement (PPI) group were invited to review the protocol and two were involved in every aspect of developing the project.
- This is a multi-centre trial, therefore the results may be more generalizable than a single centre.
- The programme is limited to English speakers only who can read at the level of an eight year-old. This may therefore exclude those who do not read in English or have low literacy.

INTRODUCTION

To effectively manage COPD, current national[1] and international[2] clinical guidelines recommend the addition of pulmonary rehabilitation (PR) to standard pharmacological therapy. PR is a non-pharmacological intervention known to help manage and improve respiratory symptoms in stable COPD conditions[3] and is also recommended following a hospital admission for acute exacerbation of COPD.[1] Moreover, PR is beneficial to improve exercise capacity,[3, 4] breathlessness,[3] quality of life,[5] anxiety and depression,[6] and reduce health care utilisation.[7] However, these benefits may only be sustained for 6 to 12 months after PR. [4] The reasons for this remain unclear and it could be linked to numerous factors, such as decrease in adherence to PR programmes, repeated exacerbations, a progression of the disease and existence of co-morbidities.[4, 8] Additionally, from a psychological perspective, patients with COPD report the feeling of abandonment after the graduation from PR as they lose the support from health care professionals and peers received during PR.[9]

Many interventions have been introduced to facilitate maintaining the benefits of PR beyond a 6 to 12 months period. Some of those studies revealed that maintenance interventions following PR is of benefit to COPD clients to maintain PR outcomes in the short and long term (6 to 24 months).[10–12] However, the interventions were heterogenous in terms of duration, setting and level of supervision following initial PR. In addition, patients'

For peer review only

motivational and behaviour change strategies play a major role in maintenance but were examined in a limited numbers of the aforementioned maintenance interventions. [10–12]

Behaviour change can be supported using motivational interviewing (MI) techniques. MI is used to help coach people to make personal changes in their behaviour.[13] MI has been shown to outperform traditional 'advice giving' in the treatment of a broad range of behavioural problems/ diseases.[14] To our knowledge, none of the previous maintenance interventions have used this technique per se. In COPD, MI coaching delivered on hospital discharge was shown to have a positive influence on readmissions and quality of life compared to a control group.[15]

Incorporating an exercise manual with group sessions underpinned by MI techniques has been tested in primary and secondary settings as a home PR alternative compared to usual care[16] and traditional (centre-based) PR. The studies revealed benefits in exercise performance, knowledge, anxiety and fatigue in which some of these outcomes were maintained 6-months after the initial delivery, when compared to usual care[16] and non-inferior to PR.[17] More recently the programme has been delivered in groups to enhance peer support in COPD. [18]

Thus, for this trial, we propose a supervised maintenance package (SPACE FOR COPD©) with the aim of retaining the expected benefits of PR as well as increasing patients' motivation and to address the feelings of abandonment following traditional PR. We will particularly focus on exacerbation and mood management, as well as promoting regular exercise in our programme. By developing this post PR intervention, we aim to preserve physical and mental well-being, potentially reducing the likelihood of repeat PR attendance and hospital readmissions. In this trial, we hypothesise that patients will have improvements in PR outcomes such as exercise capacity and overall quality of life, that will be maintained in the intervention group for at least one year. This also could be cost effective for the health service.

Aims

The overarching aim of the study is to assess whether a self-management maintenance programme (SPACE FOR COPD©), delivered on PR discharge, preserves the anticipated gains made in exercise tolerance and mental well-being at 12-months, compared to usual care alone.

For peer review only - http://bmjopen.bmj.com/site/about/guidelines.xhtml

The primary aim is:

- To compare endurance walking distance at 12-months in those who have completed the SPACE FOR COPD© maintenance programme compared to usual care.

The secondary aims are:

- To compare the following variables at 12-months in patients who have completed the SPACE FOR COPD© maintenance programme versus usual care:
  - Maximum walking distance
  - Quality of life
  - Anxiety and depression
  - Physical activity
  - Symptoms (breathlessness, fatigue, sputum production, cough, sleep, chest tightness)
  - Patient activation (knowledge, skills and confidence to manage COPD)
  - Forced Expiratory Volume in 1 second (FEV_1)
- To measure adherence to the SPACE FOR COPD© maintenance programme in terms of sessions attended, completion of the programme and manual diary entries.
- To collect data for future health economic analysis in both groups; including hospital admissions, health care contacts, personal social service costs, and patient-borne expenditure.
- To understand the patient experience of SPACE FOR COPD©; to identify facilitators and barriers of the package to maintain health and well-being.
- To work with staff, managers and commissioners involved in the delivery of SPACE FOR COPD© as a maintenance option to explore how, if successful, this programme could be rolled-out more widely.
- To use data pre-PR, where available, to evaluate the effectiveness of the initial PR programme and its impact on the maintenance rehabilitation programmes outcomes.

METHODS AND ANALYSIS

Trial design and setting

The trial will be a prospective, multicentre, randomised controlled, single blind trial of SPACE FOR COPD© maintenance programme versus usual care. There will be an integrated economic evaluation and also an embedded qualitative component. The trial will

For peer review only

be conducted at two centres, the University Hospitals of Leicester NHS Trust in Leicester, UK, and Harefield Pulmonary Rehabilitation Unit, Harefield, UK. We have utilised the SPIRIT-2013 checklist and recommendations as a guide for designing this trial.[19]

The trial will consist of three visits (baseline, six- and 12-months). Each visit will last for up to two hours where all outcomes will be collected. In addition, a two-hour small group session will be conducted at month 1 after baseline visit then at months 4, 7, and 10 in the intervention group only. See Figure 1, study flow chart. The trial recruitment will take place over one year, with a one year follow up.

Participants

Eligibility criteria for patients

Participants will be included if they meet the following criteria:

- Completed PR within the last four weeks (PR must meet standards defined by the BTS).[20]
- Clinical diagnosis of COPD, and
- Able to read and write English to the age of an eight-year-old.
- 18 years and older

Participants will be excluded if they:

- Have a significant disability which limits the daily physical activity, or
- Unable to read and write English due to the nature of the manual's language.

Figure 1

Eligibility criteria for healthcare professionals

Healthcare professionals (HCPs) involved in delivering the intervention and will be invited to participate in focus groups. The inclusion criterion is to deliver one or more sessions. The aim is to explore their experience, the barriers, and facilitators in delivering the group sessions. The HCPs are respiratory physiotherapists, respiratory nurses, or health psychologists with experience in delivering pulmonary rehabilitation. All involved HCPs have received MI training delivered by a health psychologist and introduction on how to deliver the group sessions using a dedicated trial manual.

For peer review only - http://bmjopen.bmj.com/site/about/guidelines.xhtml

Procedure

Usual Care (control group)

The control group will receive usual care: written maintenance advice as recommended by the British Thoracic Society (BTS) quality standards.[20] Leaflets will be given to signpost the individual to maintenance options for exercise following PR. A referral to community exercise schemes will be arranged for the control and intervention groups, if available or desired, as this represents best usual care across the UK.

SPACE FOR COPD© maintenance programme (intervention group)

Participants in the intervention group will receive the SPACE FOR COPD© home-based maintenance programme manual and be invited to attend SPACE FOR COPD© group-based maintenance sessions detailed below.

SPACE FOR COPD© home-based manual

Precise details of the rationale, development and efficacy of the work underpinning the SPACE FOR COPD© manual are available elsewhere.[21] The manual is divided into four stages providing an exercise program and covering educational topics. It includes self-management skills, disease specific information and tasks. The content of the manual has been approved by the Plain English Campaign and received the Crystal Mark for Clarity of British English. The content of the manual is outlined in table 1. Table 2 demonstrates the mapping of self-management components of the manual to the PRISMS taxonomy framework . There is a COPD action plan insert which is consistent with the recruiting centres' usual care.

For the purpose of this trial, a new single page insert has been developed to facilitate longer term goal setting by following the SMART (Specific, Measurable, Achievable, Relevant, and Time bound) method for goal settings. HCPs will advise participants on how to use and follow the manual independently at home. Participants will also be asked to complete section of the manual at home (e.g. exercise diaries) and will have the opportunity to call HCPs with questions via telephone.

Group sessions

The group sessions are held in small groups (5 to 10 participants per group) that will accompany the SPACE FOR COPD© maintenance programme manual with the aim of discussing progress, addressing barriers, increasing motivation to maintenance, to enhance

For peer review only

health lifestyle behaviours and to encourage social support. The content of these sessions is shown in figure 2. HCPs facilitating the groups will use MI techniques to identify barriers and facilitators to maintaining exercise, expressing empathy, supporting self-efficacy and 'rolling with resistance'. Group sessions will be facilitated by two HCPs (e.g. Physiotherapists, respiratory specialist nurses, health psychologists). There will be 4 sessions every 3 months over the period of 1 year.

We anticipate that some participants may not be able to attend all the group sessions or may be unable or unwilling to attend the research centre. In this instance, we will accommodate the participant in another session (with permission of that group) or via videoconferencing. If this isn't possible a telephone contact will be conducted instead.

Table 1. Manual components

**Stage 1**<br>What's happened to your lungs?<br>Exercise: How to get fitter<br>Setting your goals<br>Managing your stress - part 1<br>Your emotions<br>Controlling your breathing<br>Information about your medication	**Stage 4**<br>Staying fit and your hobbies<br>Your relationships<br>Dealing with setbacks<br>Sex and your lungs<br>Breathe Easy<br>Conclusion
**Stage 2**<br>How to stay fit<br>Managing days when you feel unwell<br>Saving your energy<br>Diet and feeling unwell<br>Advice for clearing your chest<br>**Stage 3**<br>How to get stronger<br>Managing your stress - part 2<br>Healthy eating<br>Travelling and your lung disease	**FAQs & Appendix**<br>Frequently asked questions<br>Setting your walking speed<br>Help for carers<br>Advice about oxygen<br>Smoking: advice on giving up<br>Cleaning your breathing devices<br>Spare walking diaries<br>Spare strength training diaries

For peer review only

Table 2. PRISMS Taxonomy components and examples in the manual

PRISMS Taxonomy Component	Elaboration	Examples from the SPACE FOR COPD© facilitator manual
**Information about condition and/ or its management**	Specific information about COPD is provided within the Stage 1 of the manual.	Participants are provided with information throughout the programme (every session)
**Information about available resources**		Participants are provided with information throughout the programme (every session)
**Provision of/agreement on specific clinical action plans and/or rescue medication**	COPD Action Plan is included as an insert in the manual	Actions plans in session 2
**Practical support with adherence (medication or behavioural)**	Walking and strength training diaries are provided for participants and discussed during sessions	Walking and strength training diaries are provided for participants and discussed during solution focused goal feedback at the beginning of sessions 2, 3 and 4
**Provision of easy access to advice or support when needed**	Participants are able to call programme facilitators between sessions with questions	Participants are provided with contact details for programme facilitators who the can call if needed (throughout programme).
**Training/rehearsal for practical self-management activities**	Including:<br>- Managing exacerbations<br>- Saving your energy	Session 2 covers Managing Exacerbations<br>Session 3 covers Saving your Energy
**Training/rehearsal for psychological strategies**	Including:<br>- Goal setting<br>- Solution focused goal feedback<br>- Problem solving<br>- Self-reward and social reward<br>- Managing stress and emotions	Goal setting activity (including action planning) and solution focused goal feedback (once every session).<br>Problem solving (throughout programme)<br>Managing stress and emotions in session 3.
**Social Support**	Including:<br>- Practical support<br>- Emotional support<br>- Peer support<br>- Group socialising is encouraged	Participants are encouraged to share experiences, advice, ideas and support each other (throughout the programme).
**Lifestyle advice and support**	Including:<br>- Introduction to the walking programme<br>- Strength training<br>- Hobbies<br>- Maintaining exercise	Introduction to walking programme in Session 1<br>Hobbies (Session 3)<br>Maintaining Exercise (Session 4)

Figure 2

Staff training and intervention fidelity

In total, eight HCPs have been trained to deliver the SPACE FOR COPD© maintenance program by a health psychologist and a specialist nurse. All attended a 1-day training course (supplemented with written material and DVD tutorials to take away) to learn the structure of the structure of the group sessions, the SPACE for COPD manual and MI skills.

The agenda covered: motivational interviewing skills, introduction to the facilitator manual and review of SPACE FOR COPD© manual, practical workshop, group facilitation, learning outcomes re-visited, and trial management. 6 of the HCPs had previously received additional MI training and all had experience in delivering PR.

A tele-conference will be held between all the facilitators across the two sites prior to and following the first intervention session to problem solve and share experiences. The tele-conference will then be continued on a regular basis to maintain the flow of the maintenance procedure and to provide peer support.

Quality Assurance will be undertaken to assess delivery of intervention content and educational style. Intervention fidelity checklists for the intervention facilitators have been adapted from a previous study.[18] One session per self-management group will be peer-observed and the checklist will be completed. Each site will observe the opposite site's group. Facilitators will complete fidelity checklists at the end of each session. These will assess: content covered, managing groups and adhering to MI principles.

For the trial, external auditing may take place at the discretion of the sponsor (UHL NHS trust).

Study outcomes

The assessments described below will be conducted at baseline, 6- and 12-month visits and completed by a blinded assessor, unaware of the group allocation. Due to the impact of COVID-19 restrictions, assessment visits and consent may be completed over the phone.

Primary outcome

Endurance capacity

The primary endpoint at 12 months is endurance capacity measured by the Endurance Shuttle Walk Test (ESWT), expressed in seconds. The ESWT is a walking field test that has been found to be safe, valid, and reproducible among patients with COPD.[23] The

For peer review only - http://bmjopen.bmj.com/site/about/guidelines.xhtml

minimal clinically important difference (MCID) for this population is estimated to be between 174 and 279 seconds.[24]

Secondary outcomes

Exercise capacity

Incremental Shuttle Walking Test (ISWT) will be used to measure the maximal exercise capacity following the test protocol published by Singh, et al.[25] The MCID in walking distance is between 35.0 and 36.1 metres.[26]

Chronic respiratory questionnaire-self reported (CRQ-SR):

CRQ-SR is a valid and reliable self-administered tool to assess health-related quality of life (HRQoL) in people with chronic lung diseases.[27] It assesses four domains with MCID defined as a mean change of 0.5 for each domain. The domains are dyspnoea (5 questions), fatigue (4 questions), emotional function (7 questions), and mastery (4 questions) and are scored on a 7-point scale per question.

Chronic obstructive pulmonary disease assessment test (CAT):

CAT is a tool developed to assess and monitor COPD symptoms and changes in patient's health status.[28] It is responsive in both short and long term assessments and is used widely in PR programmes for patients with COPD.[4] It consists of eight simple questions which cover "cough, phlegm, chest tightness, breathlessness going up hills/stairs, activity limitation at home, confidence leaving home, sleep and energy" on a 5-Likert scale. Scores ranges from 0 to 40 indicating none to very severe impact on health status.

EuroQol- five dimensions – five levels (EQ5D5L):

The EQ5D5L is a self-administered non-disease specific tool used to measure HRQoL and is developed by the EuroQol group.[29] This measures health on five dimensions (mobility, self-care, usual activities, pain or discomfort, and anxiety or depression) and a tariff is available for deriving a single utility score based on time trade-off utility scores. It also includes a visual analogue scale on which individuals rank their health today from zero (worst health) to 100 (best health). [30]

Patient activation measure:

The NHS long term plan[31] supports the engagement of patients to self-management, which is a vital key to our trial, where the 13-item patient activation measure (PAM-13) tool can be used as recommended by NHS.[32] PAM-13 tool assesses participants'

For peer review only

knowledge, skill and confidence for managing their own health and healthcare.[32] It is valid and reliable tool that defines four levels of activations ranges from low level to high level of activation.

Anxiety and depression:

The Hospital Anxiety and Depression Scale (HADS) will be used to measure anxiety and depression.[33]. It is a responsive tool and it has been tested on patients with COPD in several study settings.[34, 35] It has 14 questions to measure anxiety and depression (7 questions for each scale). Minimum and maximum scores for each scale are 0 to 21, respectively. The MCID is a mean change of 1.5 points for each scale.[35]

Physical activity monitoring:

The participants will be asked to wear a physical activity monitor after each visit (wGT3X- ActiGraph, Pensacola, FL, USA), for 7 consecutive days, except during sleeping and water-based activities. The monitor will be activated automatically for that period and doesn't require the participant to turn it on or off. It is a small device attached to an elastic belt to be worn on the waist and placed at the mid right hip. It will enable us to retrieve daily activity bouts, intensity of activities and step counts. Activity monitors can be posted out if assessment is performed over the phone.

Pulmonary function testing:

Pulmonary function will be assessed by hand-held spirometry called Vitalograph Pneumotrac 6800 (Vitalograph, Buckingham,UK) to confirm and assess the severity of COPD. The test will be performed if there was no previous test done within a three-month period. The spirometry test will be done following standard guidelines.[36] The device will be calibrated on daily basis and participants will be assessed for contraindications prior to performing the test.

Patient adherence:

The exercise diary entries embedded within the SPACE FOR COPD© manual will be used to measure adherence to the home-based maintenance programme part. Also, group session attendance will be recorded and calculated as total attendance rate. A 75 per cent cut off will be considered as a programme completion at the end of the trial. To reduce loss to follow-up we will reimburse participants' travel expenses. Where patients drop-out of the

For peer review only

SPACE FOR COPD© maintenance programme, we will still collect 6 and 12-month assessment data if consent is still in place.

Adherence to the programme is critical. To improve adherence we will be as flexible as possible with sessions and closely monitor home exercise diaries. If a participant is unable to attend a session we will offer attendance of the same session within another group (with the verbal consent of that group) or via teleconference as the preferred option or a one to one contact. We will send out text/ email/ postal reminders for group sessions and appointments. We expect that some patients may readmit to hospital and attend PR again. This will be documented but we would not deny further post-exacerbation PR in these patients, in accordance with UK guidelines.[20]

Resource use questionnaires:

Structured resource use questionnaires have been designed for the purpose of this trial and piloted prior to use. These will collect information on the number of hospital inpatient and outpatient episodes; community health & social care costs; and changes to employment status. Patient-borne costs associated with hospital visits (e.g. travel, dependant care, time off work) and out-of-pocket expenditure on products or equipment (e.g. walking shoes) will also be recorded. Questionnaires will be administered to coincide with visit times i.e. baseline, 6- and 12-months.

Interviews with patients:

Semi-structured, face-to-face interviews will be conducted with patients in the intervention group: both completers and non-completers. This will be optional and there will be a separate box on the patients' consent form for participants to opt in. The aim of the interview is to give a more complete picture of the perspective of the subjects of the study[37]; we will be adopting an interpretativist approach.[38] At the end of the 12 month period (or before if a patient decides not to continue the group sessions) we will conduct an interview to explore their experiences of using the new maintenance manual at home, the group sessions and the research process.

Participants will be recruited using a purposeful sampling technique: maximum variation sampling strategy. These interviews (n=10-20 or until data saturation) will be organised and completed by a qualitative researcher who has not been involved in delivering the intervention. Indicative interview schedules will be used and so may be

For peer review only

refined following the first 4-5 interviews. The purpose of this qualitative work is to explore perceptions of SPACE FOR COPD© as a maintenance strategy.

We will explore (Intervention) patients' experiences of SPACE FOR COPD©, identifying barriers and facilitators to the maintenance of well-being facilitated by the programme and to examine whether the sense of abandonment is lessened. The interview schedule will be flexible enough to allow completely unanticipated issues to arise. The interviewer will have adequate experience and expertise to deal with difficult situations (e.g. distress).

The patient interviews will take place at the point of trial completion (at 12-months or earlier if there is attrition). We are interested in the views of patients who do not complete the programme also. Interviews will take place either in hospital or in the patient's home or via phone or video call for those unable to attend the research centre.

Focus group for HCPs:

We will also conduct focus groups with staff involved in delivering the intervention, to understand the relative burden and benefits of this new intervention to their services. Two focus groups with 5-10 participants per group will be organised and conducted by a qualitative researcher who has not been involved in delivering the intervention.

Staff will attend a semi-structured focus group at their workplace and the findings of these will inform the training needs of those providing the intervention. They will also help to ensure that the intervention is moulded in a way that is compatible with existing models of service provision and working practices, which may help to maximise the likelihood of uptake locally and nationally.

Pre PR data

Baseline data from the initial PR will be collected, where available, to evaluate the effectiveness of the PR and its impact on the SPACE FOR COPD© maintenance programme. Data will include: ESWT, ISWT, CRQ-SR, CAT, HADS as these measures are routinely collected as part of PR.

Sample size

The power calculation (for a continuous outcome superiority trial) is based upon the primary outcome measure. We require 116 patients for an 80% chance of finding a 184 second (standard deviation: SD 282 second) difference in the ESWT time between groups at

For peer review only - http://bmjopen.bmj.com/site/about/guidelines.xhtml

12-months (at the 5% level). 184 seconds was the between group difference reported in the original primary care SPACE FOR COPD© study[16] and is similar to the suggested MCID range for this outcome (174-279 seconds).[22] This number includes expected attrition of 20 per cent.

Recruitment and invitation of participants

Participants will be approached and assessed for eligibility on completion of PR by members of the clinical PR services. Interested participants will receive a participant information sheet and given at least 48 hours to consider their participation. After consultation with our patient and public involvement (PPI) group, we propose not to set a threshold for improvement in PR outcomes for patients to be considered for the trial. Those interested will receive an invitation letter to attend a consent research visit.

Randomisation and blinding

Eligible participants will be consented and randomised using a concealed allocation web-based programme (https://www.sealedenvelope.com/). Randomisation will be 1:1 to either usual care (control) or the SPACE FOR COPD© maintenance programme (intervention). Randomisation codes will be blocked per site, providing even numbers of control and intervention patients at each site and a sufficient number of participants for the intervention group sessions to run. Due to the nature of the intervention, the participants and intervention facilitators will not be blinded to group allocation. However, the assessors will be blinded to group allocation. Other blinded assessors are in place in case of blinding exposure by first blinded assessor. Any unintentional unblinding will be documented.

Data collection and management

The REDCAP database housed by University of Leicester will be used to create our paper case report forms (CRFs) and transfer data to the electronic version will be performed periodically. This password protected database has the facility to set limits, flag discrepancies and provides an audit trail of when data has been entered/ amended and by whom. A random check of 10% of the data will be performed by a member of the research team not involved in data entry. Once data is ready for analysis, it can be easily exported to SPSS statistical analysis software. Paper CRFs will not contain patient identifiable data and

For peer review only - http://bmjopen.bmj.com/site/about/guidelines.xhtml

will be stored in locked filling cabinets in key coded offices. Data will be collected and stored in accordance with the 2018 UK General Data Protection Regulation (GDPR).

Data analysis

Quantitative analysis

All data will be analysed on an intention-to-treat basis using SPSS software (version 26.0, IBM, UK). We will conduct a separate per-protocol analysis for those who complete the intervention. Continuous variables will be presented as mean (SD), categorical variables as frequencies (percentages). All data will be assessed for normality and analysed using appropriate parametric and non-parametric statistics. To test the primary outcome, the difference between the 2 groups for the ESWT time will be analysed at 12-months using an independent t-test (or non-parametric equivalent).

Secondary outcome measures will be analysed in the same way. The analyses will be adapted to a repeated measures design to monitor changes in individual participants over time (i.e. pre-PR, post-PR, 6-months and 12-months). Significance will be set at $p<0.05$ and differences between groups will be expressed as mean (95% confidence intervals). Analysis will take into account the baseline values and adjust for potential confounders (e.g. age, disease severity). Missing data will be corrected for by multiple imputation. Interim data may be made available for abstracts but unmasking will not occur.

Economic analysis

An economic analysis will be performed from a societal perspective (including patient-borne costs) and from a health and social care perspective. Patient use of NHS services will be priced using PSSRU[39] costings or NHS reference costs[40] and include appropriate on-costs. Impact on outcomes (health utility) will be assessed using the EQ-5D questionnaire. A cost-utility analysis will compare incremental costs and marginal benefits, and include 1,000 bootstrapped replication.[41]

The cost per quality-adjusted life-year (QALY) gained, relative to standard care, will be calculated. Intervention costs will be apportioned fully to the 12-month period. A cost-effectiveness acceptability curve (CEAC) will be generated to indicate the probability that the intervention is cost-effective at various cut-off thresholds for the societal valuation of a QALY.[42] Sensitivity analyses will be undertaken.

For peer review only

Qualitative analysis

All interviews/ focus groups will be recorded and transcribed verbatim with participants' written consent. Data will be analysed according to the framework analysis approach.[43] This approach allows for data collection to start with pre-set objectives whilst ensuring that analysis reflects the experiences of participants. Framework analysis follows 5 stages: familiarisation with data, identification of thematic framework, indexing of data to thematic framework, charting of data to framework and interpretation of the framework.

Harms

Patients will be asked to complete several simple physical tests at the trial visits. They may feel breathless/ tired at the end of the walking tests but this is normal and usually settles within a few minutes. It would be very unusual for symptoms to persist. However, in this unlikely situation, medical staff are on hand at the hospital. We follow a standard operating procedure for walking tests to ensure safety. If any results from the tests undertaken as part of the trial are clinically significant, we will inform both the participant and their GP (with patient consent).

We will abide by the safeguarding policy of the University Hospitals of Leicester NHS Trust and Royal Brompton & Harefield NHS Trust to ensure that all staff working on the trial have undertaken safeguarding training.

Patient and public involvement statements

For this trial we have two PPI members on the project steering committee, who will be involved in all stages of the research cycle. We have one member from each recruiting site to ensure that local issues may be addressed. We know from previous projects how valuable our PPI colleagues can be in terms of helping to shape the research and troubleshooting any issues. For instance, our recent SPACE FOR COPD© pragmatic study (delivering the SPACE FOR COPD© programme to community groups) benefitted from PPI feedback when designing the format and scheduling of the patient group sessions to ensure that the travel arrangements and times were not onerous for patients.

For this project, PPI members have helped us to adapt the protocol in in terms of agreeing the participant inclusion criteria and how the SPACE FOR COPD© maintenance programme should be delivered. The relationship between researchers and PPI members

For peer review only

will be collaborative; the reason for this is to ensure that there is an on-going partnership between the research team and the service users, where decisions about the research are shared. To help support members we will co-produce all documentation to ensure they are written in lay language. We anticipate that the PPI members will be involved in developing and reviewing patient documents.

We would involve different stakeholders such as other health professionals, commissioners and charities (e.g. the British Lung Foundation Breathe Easy groups) in our discussions. We provide training to our PPI members and have budgeted appropriately to support this level of PPI taken from the INVOLVE guidelines.45 PPI colleagues will be involved in the dissemination of the work at a local level.

Ethics and dissemination

The trial was granted ethical approval from Health Research Authority and Health and Care Research Wales (HCRW) with Research Ethics Committee number 19/EM/0267 on 14 October 2019. The study is sponsored by University Hospitals of Leicester NHS Trust and funded by National Institute for Health Research with grant number PB-PG-0317-20032. Amendments to protocol will go through HCRW and sponsor before enforcement.

In order to have a well-structured dissemination plan, we have adopted the Scientist Knowledge Translation Plan.[44] It guides researchers through the stages of developing a dissemination strategy including clarifying 'key messages' identifying ways to transmit those messages and designing an evaluation plan. See here for details:

http://ktdrr.org/training/webcasts/webcast5/webcast_ktplan_101013r.pdf

Audiences for this trial are commissioning organisations (such as CCGs, STPs and NHS England), HCPs providing rehabilitation, patients and the public, external statutory organisations (such as the Department of Health, NICE, CLAHRCs), and Academia. They will be involved throughout the research process to better shape the outcomes and have it easily translated to the community at the end of the trial. The delivery of the trial outcomes will be communicated via face-to-face. Moreover, it will include, but not limited to, development of links with key organisations, use of electronic media, and publications including full and plain English summary reports.

For peer review only

Conclusion

PR is a highly effective intervention for patients with COPD. However the benefits are not maintained at 12-months and patients report a feeling of abandonment following the programme, with a desire for ongoing support. There is no optimal maintenance strategy following PR described in the literature or published guidelines. We have successfully tested the SPACE FOR COPD© programme in a variety of formats but never before as a maintenance intervention. In this paper, we present the protocol for a multicentre, RCT of SPACE FOR COPD© versus usual care for 12-months in patients with COPD completing PR. The primary outcome for the trial is the difference between groups at 12-months for ESWT. A number of secondary outcomes are also described.

Author Contribution

KA, CG, CN, NG, AS, WM, SS, LHW- all members of the study steering group.

The authors would like to thank Dr Claire Bourne for her assistance in developing the intervention and staff training. With thanks also to our PPI co-applicants Freda Smart (Leicester) and Chris Hedger (Harefield) and to the clinical rehabilitation teams at both sites for their assistance in refining the protocol.

Funding Statement

This work was funded by the National Institute for Health Research (NIHR) Research for Patient Benefit Programme, grant number [PB-PG-0317-20032] and supported by the NIHR Leicester Biomedical Research Centre (BRC)- Respiratory.

Figure Legend

Figure 1. Study flow chart

Figure 2. Group sessions contents

Competing interests

There are no competing interests for any author.

For peer review only

References

1 National Institute for Health and Clinical Excellence. Chronic obstructive pulmonary disease in over 16s: diagnosis and management 2018 [Available from: https://www.ncbi.nlm.nih.gov/books/NBK542426/.

2 Alison JA, McKeough ZJ, Johnston K, McNamara RJ, Spencer LM, Jenkins SC, et al. Australian and New Zealand Pulmonary Rehabilitation Guidelines. Respirology. 2017;22(4):800-19.

3 Griffiths TL, Burr ML, Campbell IA, Lewis-Jenkins V, Mullins J, Shiels K, et al. Results at 1 year of outpatient multidisciplinary pulmonary rehabilitation: a randomised controlled trial. The Lancet. 2000;355(9201):362-8.

4 Spruit MA, Singh SJ, Garvey C, ZuWallack R, Nici L, Rochester C, et al. An official American Thoracic Society/European Respiratory Society statement: key concepts and advances in pulmonary rehabilitation. Am J Respir Crit Care Med. 2013;188(8):e13-64.

5 McCarthy B, Casey D, Devane D, Murphy K, Murphy E, Lacasse Y. Pulmonary rehabilitation for chronic obstructive pulmonary disease. Cochrane Database Syst Rev. 2015(2):CD003793.

6 Harrison S, Greening N, Williams J, Morgan M, Steiner M, Singh S. Have we underestimated the efficacy of pulmonary rehabilitation in improving mood? Respiratory medicine. 2012;106(6):838-44.

7 Raskin J, Spiegler P, McCusker C, ZuWallack R, Bernstein M, Busby J, et al. The Effect of Pulmonary Rehabilitation on Healthcare Utilization in Chronic Obstructive Pulmonary Disease: THE NORTHEAST PULMONARY REHABILITATION CONSORTIUM. Journal of Cardiopulmonary Rehabilitation and Prevention. 2006;26(4):231-6.

8 Carr SJ, Goldstein RS, Brooks D. Acute exacerbations of COPD in subjects completing pulmonary rehabilitation. Chest. 2007;132(1):127-34.

9 Apps L, Barlow J, Singh S. Attitudes to exercise after a course of pulmonary rehabilitation: the patient’s perspective. 2009.

10 Beauchamp MK, Evans R, Janaudis-Ferreira T, Goldstein RS, Brooks D. Systematic review of supervised exercise programs after pulmonary rehabilitation in individuals with COPD. Chest. 2013;144(4):1124-33.

11 Berry MJ, Rejeski WJ, Adair NE, Ettinger WH, Zaccaro DJ, Sevick MA. A randomized, controlled trial comparing long-term and short-term exercise in patients with chronic obstructive pulmonary disease. Journal of Cardiopulmonary Rehabilitation and Prevention. 2003;23(1):60-8.

For peer review only

12 Meshe OF, Claydon LS, Bungay H, Andrew S. The relationship between physical activity and health status in patients with chronic obstructive pulmonary disease following pulmonary rehabilitation. Disability and rehabilitation. 2017;39(8):746-56.

13 Miller WR, Rollnick S. Motivational interviewing: Helping people change: Guilford press; 2012.

14 Rubak S, Sandbaek A, Lauritzen T, Christensen B. Motivational interviewing: a systematic review and meta-analysis. Br J Gen Pract. 2005;55(513):305-12.

15 Benzo R, Vickers K, Novotny PJ, Tucker S, Hoult J, Neuenfeldt P, et al. Health Coaching and Chronic Obstructive Pulmonary Disease Rehospitalization. A Randomized Study. Am J Respir Crit Care Med. 2016;194(6):672-80.

16 Mitchell KE, Johnson-Warrington V, Apps LD, Bankart J, Sewell L, Williams JE, et al. A self-management programme for COPD: a randomised controlled trial. Eur Respir J. 2014;44(6):1538-47.

17 Chan AW, Tetzlaff JM, Altman DG, Laupacis A, Gotzsche PC, Krleza-Jeric K, et al. SPIRIT 2013 statement: defining standard protocol items for clinical trials. Ann Intern Med. 2013;158(3):200-7.

18 Bolton CE, Bevan-Smith EF, Blakey JD, Crowe P, Elkin SL, Garrod R, et al. British Thoracic Society guideline on pulmonary rehabilitation in adults. Thorax. 2013;68 Suppl 2(Suppl 2):ii1-30.

19 Apps LD, Mitchell KE, Harrison SL, Sewell L, Williams JE, Young HM, et al. The development and pilot testing of the self-management programme of activity, coping and education for chronic obstructive pulmonary disease (SPACE for COPD). Int J Chron Obstruct Pulmon Dis. 2013;8:317-27.

20 Bourne CLA, Kanabar P, Mitchell K, Schreder S, Houchen-Wolloff L, Bankart MJG, et al. A Self-Management Programme of Activity Coping and Education - SPACE for COPD(C) - in primary care: The protocol for a pragmatic trial. Bmj Open. 2017;7(7):e014463.

21 Revill SM, Morgan MD, Singh SJ, Williams J, Hardman AE. The endurance shuttle walk: a new field test for the assessment of endurance capacity in chronic obstructive pulmonary disease. Thorax. 1999;54(3):213-22.

22 Zatloukal J, Ward S, Houchen-Wolloff L, Harvey-Dunstan T, Singh S. The minimal important difference for the endurance shuttle walk test in individuals with chronic obstructive pulmonary disease following a course of pulmonary rehabilitation. Chronic Respiratory Disease. 2019;16:1479973119853828.

For peer review only

23 Singh SJ, Morgan MD, Scott S, Walters D, Hardman AE. Development of a shuttle walking test of disability in patients with chronic airways obstruction. Thorax. 1992;47(12):1019-24.

24 Evans RA, Singh SJ. Minimum important difference of the incremental shuttle walk test distance in patients with COPD. Thorax. 2019;74(10):994-5.

25 Williams JE, Singh SJ, Sewell L, Guyatt GH, Morgan MD. Development of a self-reported Chronic Respiratory Questionnaire (CRQ-SR). Thorax. 2001;56(12):954-9.

26 Jones PW, Harding G, Berry P, Wiklund I, Chen WH, Kline Leidy N. Development and first validation of the COPD Assessment Test. Eur Respir J. 2009;34(3):648-54.

27 Cazzola M, MacNee W, Martinez F, Rabe KF, Franciosi L, Barnes P, et al. Outcomes for COPD pharmacological trials: from lung function to biomarkers. European Respiratory Journal. 2008;31(2):416-69.

28 EuroQol G. EuroQol--a new facility for the measurement of health-related quality of life. Health policy (Amsterdam, Netherlands). 1990;16(3):199.

29 Herdman M, Gudex C, Lloyd A, Janssen MF, Kind P, Parkin D, et al. Development and preliminary testing of the new five-level version of EQ-5D (EQ-5D-5L). Quality of Life Research. 2011;20(10):1727-36.

30 National Health Services. NHS Long Term Plan 2018 [Available from: https://www.longtermplan.nhs.uk/online-version/chapter-3-further-progress-on-care-quality-and-outcomes/better-care-for-major-health-conditions/respiratory-disease/.

31 National Health Services. New patient activation report launched 2014 [Available from: https://www.england.nhs.uk/2014/05/patient-activation/.

32 Hibbard JH, Mahoney ER, Stockard J, Tusler M. Development and testing of a short form of the patient activation measure. Health services research. 2005;40(6 Pt 1):1918-30.

33 Zigmond AS, Snaith RP. The hospital anxiety and depression scale. Acta Psychiatr Scand. 1983;67(6):361-70.

34 Paz-Díaz H, De Oca MM, López JM, Celli BR. Pulmonary rehabilitation improves depression, anxiety, dyspnea and health status in patients with COPD. American Journal of Physical Medicine & Rehabilitation. 2007;86(1):30-6.

35 Puhan MA, Frey M, Büchi S, Schünemann HJ. The minimal important difference of the hospital anxiety and depression scale in patients with chronic obstructive pulmonary disease. Health and quality of life outcomes. 2008;6(1):46.

For peer review only

36 British Thoracic Society. A Guide to Performing Quality Assured Diagnostic Spirometry 2013 [Available from: https://www.brit-thoracic.org.uk/media/70454/spirometry_e-guide_2013.pdf.

37 Weiss RS. Learning from strangers: The art and method of qualitative interview studies: Simon and Schuster; 1995.

38 Bryman A. Social research methods. Oxford, UK: Oxford university press; 2016.

39 Curtis L, Burns A. Unit Costs of Health and Social Care 2018 Personal Social Services Research Unit (PSSRU): University of kent, Canterbury; 2018 [Available from: http://doi.org/10.22024/UniKent/01.02.70995.

40 NHS DIGITAL. 2017-18 Reference Costs Collection 2018 [Available from: https://digital.nhs.uk/data-and-information/data-collections-and-data-sets/data-collections/reference-costs#2017-18-reference-costs-collection-.

41 O'Brien B, Briggs A. Analysis of uncertainty in health care cost-effectiveness studies: an introduction to statistical issues and methods. Statistical methods in medical research. 2002;11(6):455-68.

42 Meckley LM, Greenberg D, Cohen JT, Neumann PJ. The adoption of cost-effectiveness acceptability curves in cost-utility analyses. Med Decis Making. 2010;30(3):314-9.

43 Pope C, Ziebland S, Mays N. Qualitative research in health care: analysing qualitative data. BMJ: British Medical Journal. 2000;320(7227):114.

44 Barwick M. Developing the Knowledge Translation Plan to Build Research Impact Center on Knowledge Translation for Disability and Rehabilitation Research (KTDRR) Austin, Tx; 2013 [Available from: https://ktdrr.org/training/webcasts/webcast5/webcast_ktplan_101013r.pdf.

45 INVOLVE. Briefing notes for researchers: involving the public in NHS, public health and social care research. INVOLVE Eastleigh, UK.: NIHR; 2021 [Available from: https://www.nihr.ac.uk/documents/briefing-notes-for-researchers-public-involvement-in-nhs-health-and-social-care-research/27371

For peer review only

Figure 1. Study flow chart

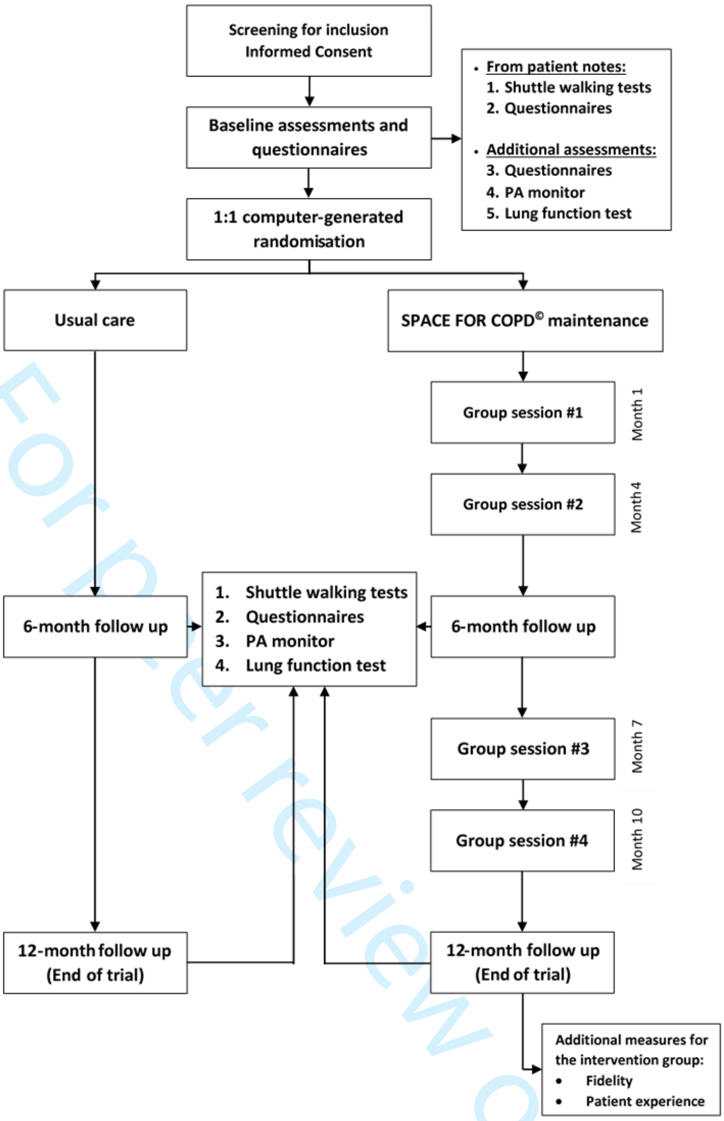

Questionnaires include: Chronic Respiratory Questionnaire-self reported, Chronic Obstructive Pulmonary Disease Assessment Test, EuroQol- five dimensions- five levels, Hospital Anxiety and Depression Scale, and Resource Use Questionnaire.

For peer review only

Figure 2. Group sessions contents

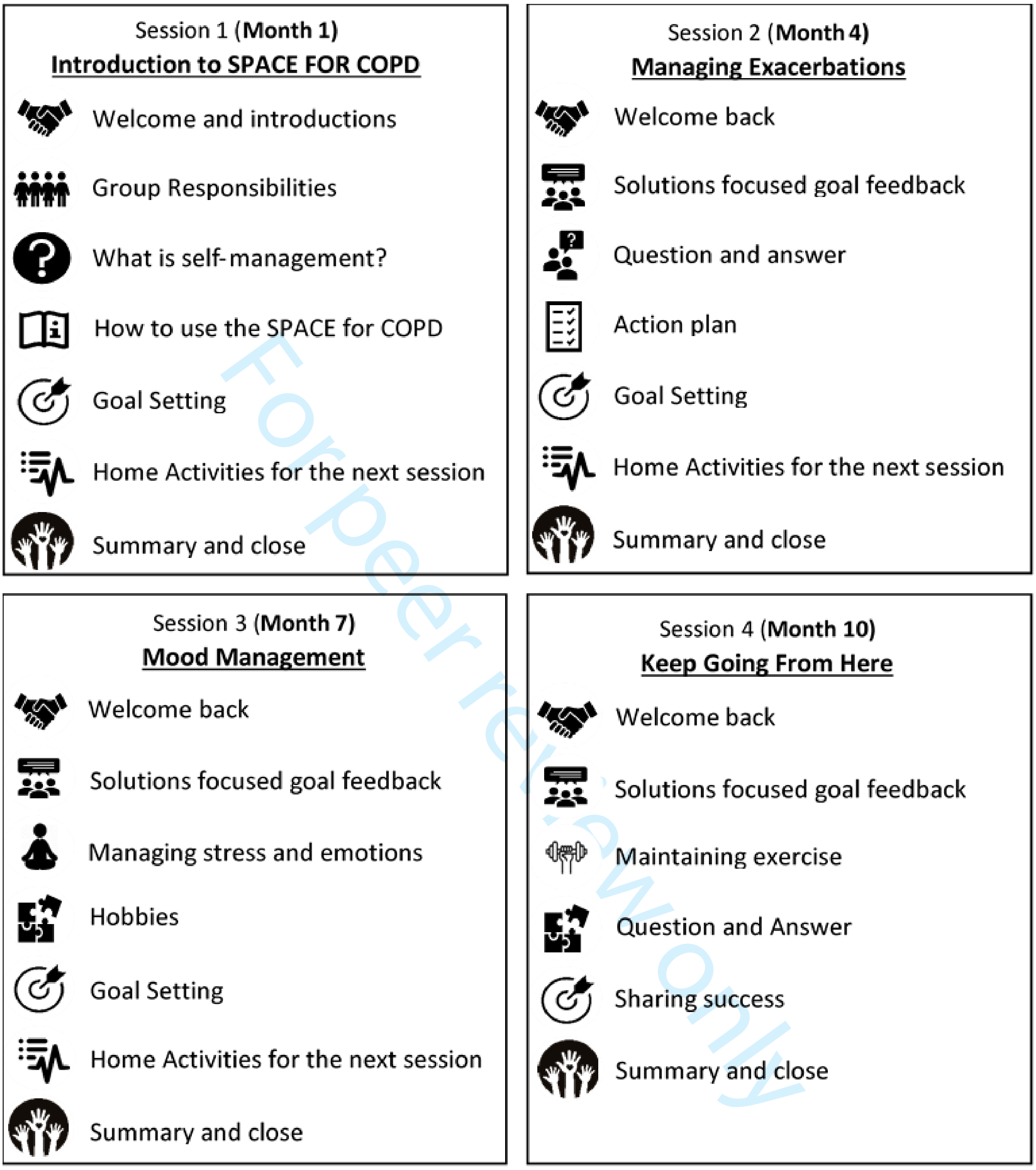

For peer review only

STANDARD PROTOCOL ITEMS: RECOMMENDATIONS FOR INTERVENTIONAL TRIALS

SPIRIT 2013 Checklist: Recommended items to address in a clinical trial protocol and related documents*

Section/item	Item No	Description	Addressed on page number
**Administrative information**			
Title	1	Descriptive title identifying the study design, population, interventions, and, if applicable, trial acronym	..........1..........
Trial registration	2a	Trial identifier and registry name. If not yet registered, name of intended registry	..........2..........
	2b	All items from the World Health Organization Trial Registration Data Set	..........NA..........
Protocol version	3	Date and version identifier	..........16..........
Funding	4	Sources and types of financial, material, and other support	..........18..........
Roles and responsibilities	5a	Names, affiliations, and roles of protocol contributors	..........18..........
	5b	Name and contact information for the trial sponsor	..........16..........
	5c	Role of study sponsor and funders, if any, in study design; collection, management, analysis, and interpretation of data; writing of the report; and the decision to submit the report for publication, including whether they will have ultimate authority over any of these activities	..........18................
	5d	Composition, roles, and responsibilities of the coordinating centre, steering committee, endpoint adjudication committee, data management team, and other individuals or groups overseeing the trial, if applicable (see Item 21a for data monitoring committee)	......NA..............

For peer review only

Introduction

Background and rationale	6a	Description of research question and justification for undertaking the trial, including summary of relevant studies (published and unpublished) examining benefits and harms for each intervention	..........2-3..........
	6b	Explanation for choice of comparators	..........3..........
Objectives	7	Specific objectives or hypotheses	..........3-4..........
Trial design	8	Description of trial design including type of trial (eg, parallel group, crossover, factorial, single group), allocation ratio, and framework (eg, superiority, equivalence, noninferiority, exploratory)	..........3..........

Methods: Participants, interventions, and outcomes

Study setting	9	Description of study settings (eg, community clinic, academic hospital) and list of countries where data will be collected. Reference to where list of study sites can be obtained	..........5..........
Eligibility criteria	10	Inclusion and exclusion criteria for participants. If applicable, eligibility criteria for study centres and individuals who will perform the interventions (eg, surgeons, psychotherapists)	..........5..........
Interventions	11a	Interventions for each group with sufficient detail to allow replication, including how and when they will be administered	..........6-7..........
	11b	Criteria for discontinuing or modifying allocated interventions for a given trial participant (eg, drug dose change in response to harms, participant request, or improving/worsening disease)	..........N/A..........
	11c	Strategies to improve adherence to intervention protocols, and any procedures for monitoring adherence (eg, drug tablet return, laboratory tests)	..........7..........
	11d	Relevant concomitant care and interventions that are permitted or prohibited during the trial	..........N/A..........
Outcomes	12	Primary, secondary, and other outcomes, including the specific measurement variable (eg, systolic blood pressure), analysis metric (eg, change from baseline, final value, time to event), method of aggregation (eg, median, proportion), and time point for each outcome. Explanation of the clinical relevance of chosen efficacy and harm outcomes is strongly recommended	..........9-13..........
Participant timeline	13	Time schedule of enrolment, interventions (including any run-ins and washouts), assessments, and visits for participants. A schematic diagram is highly recommended (see Figure)	......5, Figure 1......

For peer review only - http://bmjopen.bmj.com/site/about/guidelines.xhtml

Sample size	14	Estimated number of participants needed to achieve study objectives and how it was determined, including clinical and statistical assumptions supporting any sample size calculations	..........13..........
Recruitment	15	Strategies for achieving adequate participant enrolment to reach target sample size	..........14..........

Methods: Assignment of interventions (for controlled trials)

Allocation:

Sequence generation	16a	Method of generating the allocation sequence (eg, computer-generated random numbers), and list of any factors for stratification. To reduce predictability of a random sequence, details of any planned restriction (eg, blocking) should be provided in a separate document that is unavailable to those who enrol participants or assign interventions	..........14..........
Allocation concealment mechanism	16b	Mechanism of implementing the allocation sequence (eg, central telephone; sequentially numbered, opaque, sealed envelopes), describing any steps to conceal the sequence until interventions are assigned	..........14..........
Implementation	16c	Who will generate the allocation sequence, who will enrol participants, and who will assign participants to interventions	..........14..........
Blinding (masking)	17a	Who will be blinded after assignment to interventions (eg, trial participants, care providers, outcome assessors, data analysts), and how	..........14..........
	17b	If blinded, circumstances under which unblinding is permissible, and procedure for revealing a participant's allocated intervention during the trial	..........14..........

Methods: Data collection, management, and analysis

Data collection methods	18a	Plans for assessment and collection of outcome, baseline, and other trial data, including any related processes to promote data quality (eg, duplicate measurements, training of assessors) and a description of study instruments (eg, questionnaires, laboratory tests) along with their reliability and validity, if known. Reference to where data collection forms can be found, if not in the protocol	..........9-13 ....
	18b	Plans to promote participant retention and complete follow-up, including list of any outcome data to be collected for participants who discontinue or deviate from intervention protocols	..........11..........

For peer review only - http://bmjopen.bmj.com/site/about/guidelines.xhtml

Data management	19	Plans for data entry, coding, security, and storage, including any related processes to promote data quality (eg, double data entry; range checks for data values). Reference to where details of data management procedures can be found, if not in the protocol	..........14..........
Statistical methods	20a	Statistical methods for analysing primary and secondary outcomes. Reference to where other details of the statistical analysis plan can be found, if not in the protocol	..........15..........
	20b	Methods for any additional analyses (eg, subgroup and adjusted analyses)	..........NA..........
	20c	Definition of analysis population relating to protocol non-adherence (eg, as randomised analysis), and any statistical methods to handle missing data (eg, multiple imputation)	..........15..........
**Methods: Monitoring**			
Data monitoring	21a	Composition of data monitoring committee (DMC); summary of its role and reporting structure; statement of whether it is independent from the sponsor and competing interests; and reference to where further details about its charter can be found, if not in the protocol. Alternatively, an explanation of why a DMC is not needed	.....NA conducted by trial steering group..............
	21b	Description of any interim analyses and stopping guidelines, including who will have access to these interim results and make the final decision to terminate the trial	..........15..........
Harms	22	Plans for collecting, assessing, reporting, and managing solicited and spontaneously reported adverse events and other unintended effects of trial interventions or trial conduct	..........16..........
Auditing	23	Frequency and procedures for auditing trial conduct, if any, and whether the process will be independent from investigators and the sponsor	..........9..........
**Ethics and dissemination**			
Research ethics approval	24	Plans for seeking research ethics committee/institutional review board (REC/IRB) approval	..........16..........
Protocol amendments	25	Plans for communicating important protocol modifications (eg, changes to eligibility criteria, outcomes, analyses) to relevant parties (eg, investigators, REC/IRBs, trial participants, trial registries, journals, regulators)	..........16..........

For peer review only - http://bmjopen.bmj.com/site/about/guidelines.xhtml

Consent or assent	26a	Who will obtain informed consent or assent from potential trial participants or authorised surrogates, and how (see Item 32)	....
	26b	Additional consent provisions for collection and use of participant data and biological specimens in ancillary studies, if applicable	..........N/A..........
Confidentiality	27	How personal information about potential and enrolled participants will be collected, shared, and maintained in order to protect confidentiality before, during, and after the trial	..........14..........
Declaration of interests	28	Financial and other competing interests for principal investigators for the overall trial and each study site	........18............
Access to data	29	Statement of who will have access to the final trial dataset, and disclosure of contractual agreements that limit such access for investigators	..........15..........
Ancillary and post-trial care	30	Provisions, if any, for ancillary and post-trial care, and for compensation to those who suffer harm from trial participation	..........N/A..........
Dissemination policy	31a	Plans for investigators and sponsor to communicate trial results to participants, healthcare professionals, the public, and other relevant groups (eg, via publication, reporting in results databases, or other data sharing arrangements), including any publication restrictions	..........16-17..........
	31b	Authorship eligibility guidelines and any intended use of professional writers	.......N/A.............
	31c	Plans, if any, for granting public access to the full protocol, participant-level dataset, and statistical code	........N/A............
**Appendices**			
Informed consent materials	32	Model consent form and other related documentation given to participants and authorised surrogates	.......included as appendix.............
Biological specimens	33	Plans for collection, laboratory evaluation, and storage of biological specimens for genetic or molecular analysis in the current trial and for future use in ancillary studies, if applicable	..........N/A..........

*It is strongly recommended that this checklist be read in conjunction with the SPIRIT 2013 Explanation & Elaboration for important clarification on the items. Amendments to the protocol should be tracked and dated. The SPIRIT checklist is copyrighted by the SPIRIT Group under the Creative Commons "Attribution-NonCommercial-NoDerivs 3.0 Unported" license.

For peer review only - http://bmjopen.bmj.com/site/about/guidelines.xhtml

BMJ Open

SPACE FOR COPD© delivered as a maintenance programme on Pulmonary Rehabilitation discharge: protocol of a randomised controlled trial evaluating the long-term effects on exercise tolerance and mental wellbeing.

Journal:	*BMJ Open*
Manuscript ID	bmjopen-2021-055513.R1
Article Type:	Protocol
Date Submitted by the Author:	16-Dec-2021

Complete List of Authors:	Alqahtani, Khaled A.; Jazan University, Respiratory Therapy Department; University of Leicester, Respiratory Sciences<br>Gerlis, Charlotte; Glenfield Hospital Respiratory Medicine Department, Centre of Exercise and Rehabilitation Science, Leicester Biomedical Research Centre- Respiratory<br>Nolan, Claire; Royal Brompton and Harefield NHS Foundation Trust, Department of Respiratory Medicine<br>Gardiner, Nikki; Glenfield Hospital Respiratory Medicine Department, Centre of Exercise and Rehabilitation Science, Leicester Biomedical Research Centre- Respiratory<br>Szczepura, Ala; Coventry University, Faculty of Health and Life Sciences<br>Man, William; Royal Brompton and Harefield NHS Foundation Trust, Respiratory Medicine; Imperial College London Faculty of Medicine<br>Singh, Sally; Glenfield Hospital Respiratory Medicine Department, Centre for Exercise and Rehabilitation Science, Leicester Biomedical Research Centre-Respiratory; University of Leicester, Respiratory Sciences<br>Houchen-Wolloff, Linzy; Glenfield Hospital Respiratory Medicine Department, Centre of Exercise and Rehabilitation Science, Leicester Biomedical Research Centre- Respiratory; University of Leicester, Respiratory Sciences

**Primary Subject Heading**:	Respiratory medicine
Secondary Subject Heading:	Rehabilitation medicine, Qualitative research, Health economics
Keywords:	Protocols & guidelines < HEALTH SERVICES ADMINISTRATION & MANAGEMENT, QUALITATIVE RESEARCH, REHABILITATION MEDICINE, RESPIRATORY MEDICINE (see Thoracic Medicine), Chronic airways disease < THORACIC MEDICINE

SCHOLARONE™
Manuscripts

TITLE PAGE

Authors: Khaled Alqahtani[1,2], Charlotte Gerlis[1], Claire Nolan[3], Nikki Gardiner[1], Ala Szczepura[4], William Man[3,5], Sally J Singh[1,2], Linzy Houchen-Wolloff[1,2].

Corresponding author details: Linzy Houchen-Wolloff PhD. Address: Glenfield Hospital Respiratory Medicine Department, Centre of Exercise and Rehabilitation Science, Leicester Biomedical Research Centre- Respiratory, Leicester, UK. Telephone: 0044 0116 250 2759. Email: Linzy.Houchen@uhl-tr.nhs.uk Fax: n/a.

Co-author details:

1 Glenfield Hospital Respiratory Medicine Department, Centre of Exercise and Rehabilitation Science, Leicester Biomedical Research Centre- Respiratory, Leicester, UK.

2 University of Leicester, Respiratory Sciences, Leicester, UK.

3 Royal Brompton and Harefield NHS Foundation Trust, Respiratory Medicine, London, UK.

4 Coventry University, Faculty of Health and Life Sciences, Coventry, West Midlands, UK.

5 Imperial College London Faculty of Medicine, London, UK.

Key words: Protocols & guidelines, Rehabilitation medicine, Respiratory medicine, Chronic airways disease, Qualitative research.

Word count: 4854

TITLE

For peer review only

SPACE FOR COPD© delivered as a maintenance programme on Pulmonary Rehabilitation discharge: protocol of a randomised controlled trial evaluating the long-term effects on exercise tolerance and mental wellbeing.

ABSTRACT

Introduction The benefits achieved during pulmonary rehabilitation (PR) are known to be sustained for 6-12 months after the initial programme. Several maintenance trials have been conducted but were heterogeneous in terms of duration, frequency and labour cost. There is no consensus on one best strategy. SPACE FOR COPD© (Self-management Programme of Activity, Coping, and Education) is a home-based self-management programme which has been shown previously to be effective in primary and secondary care settings and is to be tested here as a maintenance programme. The aim is to evaluate the efficacy of the SPACE FOR COPD© programme (manual and group sessions), on exercise tolerance and mental wellbeing, compared to usual care following PR in patients with COPD.

Methods and analysis A prospective, multicentre, single-blinded randomised controlled trial requiring 116 participants with a clinical diagnosis of COPD who have finished PR within four weeks will be randomised 1:1 to either a usual care group or a SPACE FOR COPD© programme group. The intervention comprises a home-based manual and four, two-hour group sessions adopting motivational interviewing techniques over 12-months. The primary outcome is endurance capacity measured by the Endurance Shuttle Walking Test at 12 months. Secondary outcomes are; maximal exercise capacity, health-related quality of life, mood, patient activation, physical activity, lung function, and health care costs. The measures will be taken at baseline, 6- and 12-months. Patient interviews and staff focus groups will be conducted to explore barriers, facilitators, and views about the intervention at the end of the study. A framework analysis will be used for the interpretation of qualitative data.

Ethics and dissemination The trial was granted ethical approval from Health Research Authority and Health and Care Research Wales (HCRW19/EM/0267 on 14 Oct 2019). Results will be made available to all stakeholders through a dissemination event, conferences, and peer-reviewed publications.

Trial registration ISRCTN30110012.

For peer review only - http://bmjopen.bmj.com/site/about/guidelines.xhtml

STRENGTHS AND LIMITATIONS OF THIS STUDY

- This is a novel maintenance trial that uses an evidence-based home-based manual integrated with motivational interviewing skills to promote long-term self-management and behavioural change.
- Patients with COPD who are registered in our patient and public involvement (PPI) group were invited to review the protocol and two were involved in every aspect of developing the project.
- This is a multi-centre trial, therefore the results may be more generalizable than a single centre.
- The programme is limited to English speakers only who can read at the level of an eight year-old. This may therefore exclude those who do not read in English or have low literacy.

INTRODUCTION

To effectively manage COPD, current national1 and international2 clinical guidelines recommend the addition of pulmonary rehabilitation (PR) to standard pharmacological therapy. PR is a non-pharmacological intervention known to help manage and improve respiratory symptoms in stable COPD conditions3 and is also recommended following a hospital admission for acute exacerbation of COPD.1 Moreover, PR is beneficial to improve exercise capacity,3 4 breathlessness,3 quality of life (QoL),5 anxiety and depression,6 and reduce health care utilisation.7 However, these benefits may only be sustained for 6 to 12 months after PR. 4 A number of factors may affect the ability to maintain exercise capacity and QoL in the long term including: frequency of supervised maintenance exercise; strategies used to improve adherence to maintenance exercise; facilitators and barriers to long-term exercise training; and initial PR programme itself 8. Repeated exacerbations, progression of the disease and existence of co-morbidities may also have an impact4 Additionally, from a psychological perspective, patients with COPD report the feeling of abandonment after the graduation from PR as they lose the support from health care professionals and peers received during PR.9

Many interventions have been introduced to facilitate maintaining the benefits of PR beyond a 6 to 12 months period. Some of those studies revealed that maintenance interventions following PR is of benefit to COPD clients to maintain PR outcomes in the short and long term (6 to 24 months).10-12 However, the interventions were heterogenous in

For peer review only - http://bmjopen.bmj.com/site/about/guidelines.xhtml

terms of duration, setting and level of supervision following initial PR. In addition, patients' motivational and behaviour change strategies play a major role in maintenance but were examined in a limited numbers of the aforementioned maintenance interventions. 10-12

Behaviour change can be supported using motivational interviewing (MI) techniques. MI is used to help coach people to make personal changes in their behaviour.13 MI has been shown to outperform traditional 'advice giving' in the treatment of a broad range of behavioural problems/ diseases.14 To our knowledge, none of the previous maintenance interventions have used this technique per se. In COPD, MI coaching delivered on hospital discharge was shown to have a positive influence on readmissions and quality of life compared to a control group.15

Incorporating an exercise manual with group sessions underpinned by MI techniques has been tested in primary and secondary settings as a home PR alternative compared to usual care16 and traditional (centre-based) PR17. The studies revealed benefits in exercise performance, knowledge, anxiety and fatigue in which some of these outcomes were maintained 6-months after the initial delivery, when compared to usual care16 and non-inferior to PR. 17 More recently the programme has been delivered in groups to enhance peer support in COPD. 18

Thus, for this trial, we propose a supervised maintenance package (SPACE FOR COPD©) with the aim of retaining the expected benefits of PR as well as increasing patients' motivation and to address the feelings of abandonment following traditional PR. We will particularly focus on exacerbation and mood management, as well as promoting regular exercise in our programme. By developing this post PR intervention, we aim to preserve physical and mental well-being, potentially reducing the likelihood of repeat PR attendance and hospital readmissions. In this trial, we hypothesise that patients will have improvements in PR outcomes such as exercise capacity and overall quality of life that will be maintained in the intervention group for at least one year. This also could be cost effective for the health service.

Aims

The overarching aim of the study is to assess whether a self-management maintenance programme (SPACE FOR COPD©), delivered on PR discharge, preserves the anticipated gains made in exercise tolerance and mental well-being at 12-months, compared to usual care alone.

For peer review only - http://bmjopen.bmj.com/site/about/guidelines.xhtml

The primary aim is:

- To compare endurance walking distance at 12-months in those who have completed the SPACE FOR COPD© maintenance programme compared to usual care.

The secondary aims are:

- To compare the following variables at 12-months in patients who have completed the SPACE FOR COPD© maintenance programme versus usual care:
  - Maximum walking distance
  - Quality of life
  - Anxiety and depression
  - Physical activity
  - Symptoms (breathlessness, fatigue, sputum production, cough, sleep, chest tightness)
  - Patient activation (knowledge, skills and confidence to manage COPD)
  - Forced Expiratory Volume in 1 second (FEV_1)
- To measure adherence to the SPACE FOR COPD© maintenance programme in terms of sessions attended, completion of the programme and manual diary entries.
- To collect data for future health economic analysis in both groups; including hospital admissions, health care contacts, personal social service costs, and patient-borne expenditure.
- To understand the patient experience of SPACE FOR COPD©; to identify facilitators and barriers of the package to maintain health and well-being.
- To work with staff, managers and commissioners involved in the delivery of SPACE FOR COPD© as a maintenance option to explore how, if successful, this programme could be rolled-out more widely.
- To use data pre-PR, where available, to evaluate the effectiveness of the initial PR programme and its impact on the maintenance rehabilitation programmes outcomes.

METHODS AND ANALYSIS

Trial design and setting

The trial will be a prospective, multicentre, randomised controlled, single blind trial of SPACE FOR COPD© maintenance programme versus usual care. There will be an integrated economic evaluation and also an embedded qualitative component. The trial will be conducted at two centres, the University Hospitals of Leicester NHS Trust in Leicester,

For peer review only - http://bmjopen.bmj.com/site/about/guidelines.xhtml

UK, and Harefield Pulmonary Rehabilitation Unit, Harefield, UK. We have utilised the SPIRIT-2013 checklist and recommendations as a guide for designing this trial.19

The trial will consist of three visits (baseline, six- and 12-months). Each visit will last for up to two hours where all outcomes will be collected. In addition, a two-hour small group session will be conducted at month 1 after baseline visit then at months 4, 7, and 10 in the intervention group only. See Figure 1, study flow chart. The trial recruitment will take place over one year, with a one year follow up.

Participants

Eligibility criteria for patients

Participants will be included if they meet the following criteria:

- Completed PR within the last four weeks (PR must meet standards defined by the BTS). 20
- Clinical diagnosis of COPD, and
- Able to read and write English to the age of an eight-year-old.
- 18 years and older

Participants will be excluded if they:

- Have a significant disability which limits the daily physical activity, or
- Unable to read and write English due to the nature of the manual's language.

Figure 1

Eligibility criteria for healthcare professionals

Healthcare professionals (HCPs) involved in delivering the intervention and will be invited to participate in focus groups. The inclusion criterion is to deliver one or more sessions. The aim is to explore their experience, the barriers, and facilitators in delivering the group sessions. The HCPs are respiratory physiotherapists, respiratory nurses, or health psychologists with experience in delivering pulmonary rehabilitation. All involved HCPs have received MI training delivered by a health psychologist and introduction on how to deliver the group sessions using a dedicated trial manual.

For peer review only - http://bmjopen.bmj.com/site/about/guidelines.xhtml

Procedure

Usual Care (control group)

The control group will receive usual care: written maintenance advice as recommended by the British Thoracic Society (BTS) quality standards.21 Leaflets will be given to signpost the individual to maintenance options for exercise following PR. A referral to community exercise schemes will be arranged for the control and intervention groups, if available or desired, as this represents best usual care across the UK.

SPACE FOR COPD© maintenance programme (intervention group)

Participants in the intervention group will receive the SPACE FOR COPD© home-based maintenance programme manual and be invited to attend SPACE FOR COPD© group-based maintenance sessions detailed below.

SPACE FOR COPD© home-based manual

Precise details of the rationale, development and efficacy of the work underpinning the SPACE FOR COPD© manual are available elsewhere.22 The manual is divided into four stages providing an exercise program and covering educational topics. It includes self-management skills, disease specific information and tasks. The content of the manual has been approved by the Plain English Campaign and received the Crystal Mark for Clarity of British English. The content of the manual is outlined in table 1. Table 2 demonstrates the mapping of self-management components of the manual to the PRISMS taxonomy framework. There is a COPD action plan insert which is consistent with the recruiting centres' usual care.

For the purpose of this trial, a new single page insert has been developed to facilitate longer term goal setting by following the SMART (Specific, Measurable, Achievable, Relevant, and Time bound) method for goal settings. HCPs will advise participants on how to use and follow the manual independently at home. Participants will also be asked to complete section of the manual at home (e.g. exercise diaries) and will have the opportunity to call HCPs with questions via telephone.

Group sessions

The group sessions are held in small groups (5 to 10 participants per group) that will accompany the SPACE FOR COPD© maintenance programme manual with the aim of discussing progress, addressing barriers, increasing motivation to maintenance, to enhance health lifestyle behaviours and to encourage social support. The content of these sessions is shown in figure 2. HCPs facilitating the groups will use MI techniques to identify barriers and

For peer review only - http://bmjopen.bmj.com/site/about/guidelines.xhtml

facilitators to maintaining exercise, expressing empathy, supporting self-efficacy and 'rolling with resistance'. Group sessions will be facilitated by two HCPs (e.g. Physiotherapists, respiratory specialist nurses, health psychologists). There will be 4 sessions every 3 months over the period of 1 year.

We anticipate that some participants may not be able to attend all the group sessions or may be unable or unwilling to attend the research centre. In this instance, we will accommodate the participant in another session (with permission of that group) or via videoconferencing. If this isn't possible a telephone contact will be conducted instead.

Table 1. Manual components

**Stage 1**<br>What's happened to your lungs?<br>Exercise: How to get fitter<br>Setting your goals<br>Managing your stress - part 1<br>Your emotions<br>Controlling your breathing<br>Information about your medication	**Stage 4**<br>Staying fit and your hobbies<br>Your relationships<br>Dealing with setbacks<br>Sex and your lungs<br>Breathe Easy<br>Conclusion
**Stage 2**<br>How to stay fit<br>Managing days when you feel unwell<br>Saving your energy<br>Diet and feeling unwell<br>Advice for clearing your chest<br>**Stage 3**<br>How to get stronger<br>Managing your stress - part 2<br>Healthy eating<br>Travelling and your lung disease	**FAQs & Appendix**<br>Frequently asked questions<br>Setting your walking speed<br>Help for carers<br>Advice about oxygen<br>Smoking: advice on giving up<br>Cleaning your breathing devices<br>Spare walking diaries<br>Spare strength training diaries

For peer review only

Table 2. PRISMS Taxonomy components and examples in the manual

PRISMS Taxonomy Component	Elaboration	Examples from the SPACE FOR COPD© facilitator manual
**Information about condition and/ or its management**	Specific information about COPD is provided within the Stage 1 of the manual.	Participants are provided with information throughout the programme (every session)
**Information about available resources**		Participants are provided with information throughout the programme (every session)
**Provision of/agreement on specific clinical action plans and/or rescue medication**	COPD Action Plan is included as an insert in the manual	Actions plans in session 2
**Practical support with adherence (medication or behavioural)**	Walking and strength training diaries are provided for participants and discussed during sessions	Walking and strength training diaries are provided for participants and discussed during solution focused goal feedback at the beginning of sessions 2, 3 and 4
**Provision of easy access to advice or support when needed**	Participants are able to call programme facilitators between sessions with questions	Participants are provided with contact details for programme facilitators who the can call if needed (throughout programme).
**Training/rehearsal for practical self-management activities**	Including:<br>- Managing exacerbations<br>- Saving your energy	Session 2 covers Managing Exacerbations<br>Session 3 covers Saving your Energy
**Training/rehearsal for psychological strategies**	Including:<br>- Goal setting<br>- Solution focused goal feedback<br>- Problem solving<br>- Self-reward and social reward<br>- Managing stress and emotions	Goal setting activity (including action planning) and solution focused goal feedback (once every session).<br>Problem solving (throughout programme)<br>Managing stress and emotions in session 3.
**Social Support**	Including:<br>- Practical support<br>- Emotional support<br>- Peer support<br>- Group socialising is encouraged	Participants are encouraged to share experiences, advice, ideas and support each other (throughout the programme).
**Lifestyle advice and support**	Including:<br>- Introduction to the walking programme<br>- Strength training	Introduction to walking programme in Session 1<br>Hobbies (Session 3)<br>Maintaining Exercise (Session 4)

For peer review only - http://bmjopen.bmj.com/site/about/guidelines.xhtml

	- Hobbies<br>- Maintaining exercise	

Figure 2

Staff training and intervention fidelity

In total, eight HCPs have been trained to deliver the SPACE FOR COPD© maintenance program by a health psychologist and a specialist nurse. All attended a 1-day training course (supplemented with written material and DVD tutorials to take away) to learn the structure of the structure of the group sessions, the SPACE for COPD manual and MI skills.

The agenda covered: motivational interviewing skills, introduction to the facilitator manual and review of SPACE FOR COPD© manual, practical workshop, group facilitation, learning outcomes re-visited, and trial management. 6 of the HCPs had previously received additional MI training and all had experience in delivering PR.

A tele-conference will be held between all the facilitators across the two sites prior to and following the first intervention session to problem solve and share experiences. The tele-conference will then be continued on a regular basis to maintain the flow of the maintenance procedure and to provide peer support.

Quality Assurance will be undertaken to assess delivery of intervention content and educational style. Intervention fidelity checklists for the intervention facilitators have been adapted from a previous study.[18] One session per self-management group will be peer-observed and the checklist will be completed. Each site will observe the opposite site's group. Facilitators will complete fidelity checklists at the end of each session. These will assess: content covered, managing groups and adhering to MI principles.

For the trial, external auditing may take place at the discretion of the sponsor (UHL NHS trust).

Study outcomes

The assessments described below will be conducted at baseline, 6- and 12-month visits and completed by a blinded assessor, unaware of the group allocation. Due to the impact of COVID-19 restrictions, assessment visits and consent may be completed over the phone for all data other than physical tests where this is not possible (i.e. spirometry/ walking tests).

For peer review only

Primary outcome

Endurance capacity

The primary endpoint at 12 months is endurance capacity measured by the Endurance Shuttle Walk Test (ESWT), expressed in seconds. The ESWT is a walking field test that has been found to be safe, valid, and reproducible among patients with COPD.23 The minimal clinically important difference (MCID) for this population is estimated to be between 174 and 279 seconds.24

Secondary outcomes

Exercise capacity

Incremental Shuttle Walking Test (ISWT) will be used to measure the maximal exercise capacity following the test protocol published by Singh, et al.25 The MCID in walking distance is between 35.0 and 36.1 metres.26

Chronic respiratory questionnaire-self reported (CRQ-SR):

CRQ-SR is a valid and reliable self-administered tool to assess health-related quality of life (HRQoL) in people with chronic lung diseases.27 It assesses four domains with MCID defined as a mean change of 0.5 for each domain. The domains are dyspnoea (5 questions), fatigue (4 questions), emotional function (7 questions), and mastery (4 questions) and are scored on a 7-point scale per question.

Chronic obstructive pulmonary disease assessment test (CAT):

CAT is a tool developed to assess and monitor COPD symptoms and changes in patient's health status.28 It is responsive in both short and long term assessments and is used widely in PR programmes for patients with COPD.4 It consists of eight simple questions which cover "cough, phlegm, chest tightness, breathlessness going up hills/stairs, activity limitation at home, confidence leaving home, sleep and energy" on a 5-Likert scale. Scores ranges from 0 to 40 indicating none to very severe impact on health status.

EuroQol- five dimensions – five levels (EQ5D5L):

The EQ5D5L is a self-administered non-disease specific tool used to measure HRQoL and is developed by the EuroQol group.29 This measures health on five dimensions (mobility, self-care, usual activities, pain or discomfort, and anxiety or depression) and a tariff is available for deriving a single utility score based on time trade-off utility scores. It also includes a visual analogue scale on which individuals rank their health today from zero (worst health) to 100 (best health). 30

For peer review only

Patient activation measure:

The NHS long term plan31 supports the engagement of patients to self-management, which is a vital key to our trial, where the 13-item patient activation measure (PAM-13) tool can be used as recommended by NHS.32 PAM-13 tool assesses participants' knowledge, skill and confidence for managing their own health and healthcare.33 It is valid and reliable tool that defines four levels of activations ranges from low level to high level of activation.

Anxiety and depression:

The Hospital Anxiety and Depression Scale (HADS) will be used to measure anxiety and depression.34. It is a responsive tool and it has been tested on patients with COPD in several study settings.35 It has 14 questions to measure anxiety and depression (7 questions for each scale). Minimum and maximum scores for each scale are 0 to 21, respectively. The MCID is a mean change of 1.5 points for each scale.36

Physical activity monitoring:

The participants will be asked to wear a physical activity monitor after each visit (wGT3X- ActiGraph, Pensacola, FL, USA), for 7 consecutive days, except during sleeping and water-based activities. The monitor will be activated automatically for that period and doesn't require the participant to turn it on or off. It is a small device attached to an elastic belt to be worn on the waist and placed at the mid right hip. It will enable us to retrieve daily activity bouts, intensity of activities and step counts. Activity monitors can be posted out if assessment is performed over the phone.

Pulmonary function testing:

Pulmonary function will be assessed by hand-held spirometry called Vitalograph Pneumotrac 6800 (Vitalograph, Buckingham,UK) to confirm and assess the severity of COPD. The test will be performed if there was no previous test done within a three-month period. The spirometry test will be done following standard guidelines.37 The device will be calibrated on daily basis and participants will be assessed for contraindications prior to performing the test.

Patient adherence:

The exercise diary entries embedded within the SPACE FOR COPD© manual will be used to measure adherence to the home-based maintenance programme part. Also, group session attendance will be recorded and calculated as total attendance rate. A 75 per cent

For peer review only - http://bmjopen.bmj.com/site/about/guidelines.xhtml

cut off will be considered as a programme completion at the end of the trial. To reduce loss to follow-up we will reimburse participants' travel expenses. Where patients drop-out of the SPACE FOR COPD© maintenance programme, we will still collect 6 and 12-month assessment data if consent is still in place.

Adherence to the programme is critical. To improve adherence we will be as flexible as possible with sessions and closely monitor home exercise diaries. If a participant is unable to attend a session we will offer attendance of the same session within another group (with the verbal consent of that group) or via teleconference as the preferred option or a one to one contact. We will send out text/ email/ postal reminders for group sessions and appointments. We expect that some patients may readmit to hospital and attend PR again. This will be documented but we would not deny further post-exacerbation PR in these patients, in accordance with UK guidelines.[20]

Resource use questionnaires:

Structured resource use questionnaires have been designed for the purpose of this trial and piloted prior to use. These will collect information on the number of hospital inpatient and outpatient episodes; community health & social care costs; and changes to employment status. Patient-borne costs associated with hospital visits (e.g. travel, dependant care, time off work) and out-of-pocket expenditure on products or equipment (e.g. walking shoes) will also be recorded. Questionnaires will be administered to coincide with visit times i.e. baseline, 6- and 12-months.

Interviews with patients:

Semi-structured, face-to-face interviews will be conducted with patients in the intervention group: both completers and non-completers. This will be optional and there will be a separate box on the patients' consent form for participants to opt in. The aim of the interview is to give a more complete picture of the perspective of the subjects of the study[38]; we will be adopting an interpretativist approach.[39] At the end of the 12 month period (or before if a patient decides not to continue the group sessions) we will conduct an interview to explore their experiences of using the new maintenance manual at home, the group sessions and the research process.

Participants will be recruited using a purposeful sampling technique: maximum variation sampling strategy. These interviews (n=10-20 or until data saturation) will be organised and completed by a qualitative researcher who has not been involved in

For peer review only - http://bmjopen.bmj.com/site/about/guidelines.xhtml

delivering the intervention. Indicative interview schedules will be used and so may be refined following the first 4-5 interviews. The purpose of this qualitative work is to explore perceptions of SPACE FOR COPD© as a maintenance strategy.

We will explore (Intervention) patients' experiences of SPACE FOR COPD©, identifying barriers and facilitators to the maintenance of well-being facilitated by the programme and to examine whether the sense of abandonment is lessened. The interview schedule will be flexible enough to allow completely unanticipated issues to arise. The interviewer will have adequate experience and expertise to deal with difficult situations (e.g. distress).

The patient interviews will take place at the point of trial completion (at 12-months or earlier if there is attrition). We are interested in the views of patients who do not complete the programme also. Interviews will take place either in hospital or in the patient's home or via phone or video call for those unable to attend the research centre.

Focus group for HCPs:

We will also conduct focus groups with staff involved in delivering the intervention, to understand the relative burden and benefits of this new intervention to their services. Two focus groups with 5-10 participants per group will be organised and conducted by a qualitative researcher who has not been involved in delivering the intervention.

Staff will attend a semi-structured focus group at their workplace and the findings of these will inform the training needs of those providing the intervention. They will also help to ensure that the intervention is moulded in a way that is compatible with existing models of service provision and working practices, which may help to maximise the likelihood of uptake locally and nationally.

Pre PR data

Baseline data from the initial PR will be collected, where available, to evaluate the effectiveness of the PR and its impact on the SPACE FOR COPD© maintenance programme. Data will include: ESWT, ISWT, CRQ-SR, CAT, HADS as these measures are routinely collected as part of PR.

Sample size

The power calculation (for a continuous outcome superiority trial) is based upon the primary outcome measure. We require 116 patients for an 80% chance of finding a 184 second (standard deviation: SD 282 second) difference in the ESWT time between groups at

For peer review only

12-months (at the 5% level). 184 seconds was the between group difference reported in the original primary care SPACE FOR COPD© study16 and is similar to the suggested MCID range for this outcome (174-279 seconds).24 This number includes expected attrition of 20 per cent.

Recruitment and invitation of participants

Participants will be approached and assessed for eligibility on completion of PR by members of the clinical PR services. Interested participants will receive a participant information sheet and given at least 48 hours to consider their participation. After consultation with our patient and public involvement (PPI) group, we propose not to set a threshold for improvement in PR outcomes for patients to be considered for the trial. Those interested will receive an invitation letter to attend a consent research visit.

Randomisation and blinding

Eligible participants will be consented and randomised using a concealed allocation web-based programme (https://www.sealedenvelope.com/). Randomisation will be 1:1 to either usual care (control) or the SPACE FOR COPD© maintenance programme (intervention). Randomisation codes will be blocked per site, providing even numbers of control and intervention patients at each site and a sufficient number of participants for the intervention group sessions to run. Due to the nature of the intervention, the participants and intervention facilitators will not be blinded to group allocation. However, the assessors will be blinded to group allocation. Other blinded assessors are in place in case of blinding exposure by first blinded assessor. Any unintentional unblinding will be documented.

Data collection and management

The REDCAP database housed by University of Leicester will be used to create our paper case report forms (CRFs) and transfer data to the electronic version will be performed periodically. This password protected database has the facility to set limits, flag discrepancies and provides an audit trail of when data has been entered/ amended and by whom. A random check of 10% of the data will be performed by a member of the research team not involved in data entry. Once data is ready for analysis, it can be easily exported to SPSS statistical analysis software. Paper CRFs will not contain patient identifiable data and

For peer review only - http://bmjopen.bmj.com/site/about/guidelines.xhtml

will be stored in locked filling cabinets in key coded offices. Data will be collected and stored in accordance with the 2018 UK General Data Protection Regulation (GDPR).

Data analysis

Quantitative analysis

All data will be analysed on an intention-to-treat basis using SPSS software (version 26.0, IBM, UK). We will conduct a separate per-protocol analysis for those who complete the intervention. Continuous variables will be presented as mean (SD), categorical variables as frequencies (percentages). All data will be assessed for outliers, normality and analysed using appropriate parametric and non-parametric statistics. To test the primary outcome, the difference between the 2 groups for the ESWT time will be analysed at 12-months using an independent t-test (or non-parametric equivalent).

Secondary outcome measures will be analysed in the same way. We will also look at changes in all outcomes in individual participants over time (i.e. pre-PR, post-PR, 6-months and 12-months) using a repeated measures ANOVA (or non-parametric alternative). Significance will be set at $p<0.05$ and differences between groups will be expressed as mean (95% confidence intervals). Analysis will take into account the baseline values and adjust for potential confounders (e.g. age, disease severity). Missing data will be corrected for by multiple imputation. Interim data may be made available for abstracts but unmasking will not occur.

Economic analysis

An economic analysis will be performed from a societal perspective (including patient-borne costs) and from a health and social care perspective. Patient use of NHS services will be priced using PSSRU[40] costings or NHS reference costs[41] and include appropriate on-costs. Impact on outcomes (health utility) will be assessed using the EQ-5D questionnaire. A cost-utility analysis will compare incremental costs and marginal benefits, and include 1,000 bootstrapped replication.[42.]

The cost per quality-adjusted life-year (QALY) gained, relative to standard care, will be calculated. Intervention costs will be apportioned fully to the 12-month period. A cost-effectiveness acceptability curve (CEAC) will be generated to indicate the probability that the intervention is cost-effective at various cut-off thresholds for the societal valuation of a QALY.[43] Sensitivity analyses will be undertaken.

For peer review only - http://bmjopen.bmj.com/site/about/guidelines.xhtml

Qualitative analysis

All interviews/ focus groups will be recorded and transcribed verbatim with participants' written consent. Data will be analysed according to the framework analysis approach.44 This approach allows for data collection to start with pre-set objectives whilst ensuring that analysis reflects the experiences of participants. Framework analysis follows 5 stages: familiarisation with data, identification of thematic framework, indexing of data to thematic framework, charting of data to framework and interpretation of the framework.

Harms

Patients will be asked to complete several simple physical tests at the trial visits. They may feel breathless/ tired at the end of the walking tests but this is normal and usually settles within a few minutes. It would be very unusual for symptoms to persist. However, in this unlikely situation, medical staff are on hand at the hospital. We follow a standard operating procedure for walking tests to ensure safety. If any results from the tests undertaken as part of the trial are clinically significant, we will inform both the participant and their GP (with patient consent).

We will abide by the safeguarding policy of the University Hospitals of Leicester NHS Trust and Royal Brompton & Harefield NHS Trust to ensure that all staff working on the trial have undertaken safeguarding training.

Patient and public involvement statements

For this trial we have two PPI members on the project steering committee, who will be involved in all stages of the research cycle. We have one member from each recruiting site to ensure that local issues may be addressed. We know from previous projects how valuable our PPI colleagues can be in terms of helping to shape the research and troubleshooting any issues. For instance, our recent SPACE FOR COPD© pragmatic study (delivering the SPACE FOR COPD© programme to community groups) benefitted from PPI feedback when designing the format and scheduling of the patient group sessions to ensure that the travel arrangements and times were not onerous for patients.

For this project, PPI members have helped us to adapt the protocol in in terms of agreeing the participant inclusion criteria and how the SPACE FOR COPD© maintenance programme should be delivered. The relationship between researchers and PPI members will be collaborative; the reason for this is to ensure that there is an on-going partnership

For peer review only - http://bmjopen.bmj.com/site/about/guidelines.xhtml

between the research team and the service users, where decisions about the research are shared. To help support members we will co-produce all documentation to ensure they are written in lay language. We anticipate that the PPI members will be involved in developing and reviewing patient documents.

We would involve different stakeholders such as other health professionals, commissioners and charities (e.g. the British Lung Foundation Breathe Easy groups) in our discussions. We provide training to our PPI members and have budgeted appropriately to support this level of PPI taken from the INVOLVE guidelines.[45] PPI colleagues will be involved in the dissemination of the work at a local level.

Ethics and dissemination

The trial was granted ethical approval from Health Research Authority and Health and Care Research Wales (HCRW) with Research Ethics Committee number 19/EM/0267 on 14th October 2019. The study is sponsored by University Hospitals of Leicester NHS Trust and funded by National Institute for Health Research with grant number PB-PG-0317-20032. Amendments to protocol will go through HCRW and sponsor before enforcement.

In order to have a well-structured dissemination plan, we have adopted the Scientist Knowledge Translation Plan.[46] It guides researchers through the stages of developing a dissemination strategy including clarifying 'key messages' identifying ways to transmit those messages and designing an evaluation plan. See here for details: http://ktdrr.org/training/webcasts/webcast5/webcast_ktplan_101013r.pdf

Audiences for this trial are commissioning organisations (such as CCGs, STPs and NHS England), HCPs providing rehabilitation, patients and the public, external statutory organisations (such as the Department of Health, NICE, CLAHRCs), and Academia. They will be involved throughout the research process to better shape the outcomes and have it easily translated to the community at the end of the trial. The delivery of the trial outcomes will be communicated via face-to-face. Moreover, it will include, but not limited to, development of links with key organisations, use of electronic media, and publications including full and plain English summary reports.

For peer review only

Author Contribution

KA, CG, CN, NG, AS, WM, SS, LHW- all members of the study steering group.

The authors would like to thank Dr Claire Bourne for her assistance in developing the intervention and staff training. With thanks also to our PPI co-applicants Freda Smart (Leicester) and Chris Hedger (Harefield) and to the clinical rehabilitation teams at both sites for their assistance in refining the protocol.

Funding Statement

This work was funded by the National Institute for Health Research (NIHR) Research for Patient Benefit Programme, grant number [PB-PG-0317-20032] and supported by the NIHR Leicester Biomedical Research Centre (BRC)- Respiratory.

Figure Legend

Figure 1. Study flow chart

Figure 2. Group sessions contents

Competing interests

There are no competing interests for any author.

For peer review only

References

1 National Institute for Health and Clinical Excellence. Chronic obstructive pulmonary disease in over 16s: diagnosis and management 2018 [Available from: https://www.ncbi.nlm.nih.gov/books/NBK542426/.

2 Alison JA, McKeough ZJ, Johnston K, McNamara RJ, Spencer LM, Jenkins SC, et al. Australian and New Zealand Pulmonary Rehabilitation Guidelines. Respirology. 2017;22(4):800-19.

3 Griffiths TL, Burr ML, Campbell IA, Lewis-Jenkins V, Mullins J, Shiels K, et al. Results at 1 year of outpatient multidisciplinary pulmonary rehabilitation: a randomised controlled trial. The Lancet. 2000;355(9201):362-8.

4 Spruit MA, Singh SJ, Garvey C, ZuWallack R, Nici L, Rochester C, et al. An official American Thoracic Society/European Respiratory Society statement: key concepts and advances in pulmonary rehabilitation. Am J Respir Crit Care Med. 2013;188(8):e13-64.

5 McCarthy B, Casey D, Devane D, Murphy K, Murphy E, Lacasse Y. Pulmonary rehabilitation for chronic obstructive pulmonary disease. Cochrane Database Syst Rev. 2015(2):CD003793.

6 Harrison S, Greening N, Williams J, Morgan M, Steiner M, Singh S. Have we underestimated the efficacy of pulmonary rehabilitation in improving mood? Respiratory medicine. 2012;106(6):838-44.

7 Raskin J, Spiegler P, McCusker C, ZuWallack R, Bernstein M, Busby J, et al. The Effect of Pulmonary Rehabilitation on Healthcare Utilization in Chronic Obstructive Pulmonary Disease: THE NORTHEAST PULMONARY REHABILITATION CONSORTIUM. Journal of Cardiopulmonary Rehabilitation and Prevention. 2006;26(4):231-6.

8 Spencer, LM, McKeough, ZJ. Maintaining the benefits following pulmonary rehabilitation: Achievable or not? Respirology. 2019; 24: 909– 915.9 Apps L, Barlow J, Singh S. Attitudes to exercise after a course of pulmonary rehabilitation: the patient's perspective. 2009.

10 Beauchamp MK, Evans R, Janaudis-Ferreira T, Goldstein RS, Brooks D. Systematic review of supervised exercise programs after pulmonary rehabilitation in individuals with COPD. Chest. 2013;144(4):1124-33.

11 Jenkins AR, Gowler H, Curtis F, Holden NS, Bridle C, Jones AW. Efficacy of supervised maintenance exercise following pulmonary rehabilitation on health care use: a systematic review and meta-analysis. Int J COPD. 2018;13:257–73.

12 Malaguti C, Dal Corso S, Janjua S, Holland AE. Supervised maintenance programmes following pulmonary rehabilitation compared to usual care for chronic obstructive pulmonary disease. Cochrane Database of Systematic Reviews 2021, Issue 8. Art. No.: CD013569.

13 Miller WR, Rollnick S. Motivational interviewing: Helping people change: Guilford press; 2012.

14 Rubak S, Sandbaek A, Lauritzen T, Christensen B. Motivational interviewing: a systematic review and meta-analysis. Br J Gen Pract. 2005;55(513):305-12.

15 Benzo R, Vickers K, Novotny PJ, Tucker S, Hoult J, Neuenfeldt P, et al. Health Coaching and Chronic Obstructive Pulmonary Disease Rehospitalization. A Randomized Study. Am J Respir Crit Care Med. 2016;194(6):672-80.

For peer review only - http://bmjopen.bmj.com/site/about/guidelines.xhtml

16 Mitchell KE, Johnson-Warrington V, Apps LD, Bankart J, Sewell L, Williams JE, et al. A self-management programme for COPD: a randomised controlled trial. Eur Respir J. 2014;44(6):1538-47.

17 Horton EJ, Mitchell KE, Johnson-Warrington V, et alComparison of a structured home-based rehabilitation programme with conventional supervised pulmonary rehabilitation: a randomised non-inferiority trialThorax 2018;73:29-36.

18 Bourne CLA, Kanabar P, Mitchell K, Schreder S, Houchen-Wolloff L, Bankart MJG, et al. A Self-Management Programme of Activity Coping and Education - SPACE for COPD(C) - in primary care: The protocol for a pragmatic trial. Bmj Open. 2017;7(7):e014463.

19 Chan AW, Tetzlaff JM, Altman DG, Laupacis A, Gotzsche PC, Krleza-Jeric K, et al. SPIRIT 2013 statement: defining standard protocol items for clinical trials. Ann Intern Med. 2013;158(3):200-7.

20 Bolton CE, Bevan-Smith EF, Blakey JD, Crowe P, Elkin SL, Garrod R, et al. British Thoracic Society guideline on pulmonary rehabilitation in adults. Thorax. 2013;68 Suppl 2(Suppl 2):ii1-30.

21 BTS Pulmonary rehabilitation quality standards. Published by: British Thoracic Society. Publication date: May 2014. Accessible here: https://www.brit-thoracic.org.uk/quality-improvement/quality-standards/pulmonary-rehabilitation/ Last accessed: 15/12/21.

22 Apps LD, Mitchell KE, Harrison SL, Sewell L, Williams JE, Young HM, et al. The development and pilot testing of the self-management programme of activity, coping and education for chronic obstructive pulmonary disease (SPACE for COPD). Int J Chron Obstruct Pulmon Dis. 2013;8:317-27.

23 Revill SM, Morgan MD, Singh SJ, Williams J, Hardman AE. The endurance shuttle walk: a new field test for the assessment of endurance capacity in chronic obstructive pulmonary disease. Thorax. 1999;54(3):213-22.

24 Zatloukal J, Ward S, Houchen-Wolloff L, Harvey-Dunstan T, Singh S. The minimal important difference for the endurance shuttle walk test in individuals with chronic obstructive pulmonary disease following a course of pulmonary rehabilitation. Chronic Respiratory Disease. 2019;16:1479973119853828.

25 Singh SJ, Morgan MD, Scott S, Walters D, Hardman AE. Development of a shuttle walking test of disability in patients with chronic airways obstruction. Thorax. 1992;47(12):1019-24.

26 Evans RA, Singh SJ. Minimum important difference of the incremental shuttle walk test distance in patients with COPD. Thorax. 2019;74(10):994-5.

For peer review only

27 Williams JE, Singh SJ, Sewell L, Guyatt GH, Morgan MD. Development of a self-reported Chronic Respiratory Questionnaire (CRQ-SR). Thorax. 2001;56(12):954-9.

28 Jones PW, Harding G, Berry P, Wiklund I, Chen WH, Kline Leidy N. Development and first validation of the COPD Assessment Test. Eur Respir J. 2009;34(3):648-54.

29 Cazzola M, MacNee W, Martinez F, Rabe KF, Franciosi L, Barnes P, et al. Outcomes for COPD pharmacological trials: from lung function to biomarkers. European Respiratory Journal. 2008;31(2):416-69.

30 EuroQol G. EuroQol--a new facility for the measurement of health-related quality of life. Health policy (Amsterdam, Netherlands). 1990;16(3):199.

31 National Health Services. NHS Long Term Plan 2018 [Available from: https://www.longtermplan.nhs.uk/online-version/chapter-3-further-progress-on-care-quality-and-outcomes/better-care-for-major-health-conditions/respiratory-disease/.

32 National Health Services. New patient activation report launched 2014 [Available from: https://www.england.nhs.uk/2014/05/patient-activation/.

33 Hibbard JH, Mahoney ER, Stockard J, Tusler M. Development and testing of a short form of the patient activation measure. Health services research. 2005;40(6 Pt 1):1918-30.

34 Zigmond AS, Snaith RP. The hospital anxiety and depression scale. Acta Psychiatr Scand. 1983;67(6):361-70.

35 Paz-Díaz H, De Oca MM, López JM, Celli BR. Pulmonary rehabilitation improves depression, anxiety, dyspnea and health status in patients with COPD. American Journal of Physical Medicine & Rehabilitation. 2007;86(1):30-6.

36 Puhan MA, Frey M, Büchi S, Schünemann HJ. The minimal important difference of the hospital anxiety and depression scale in patients with chronic obstructive pulmonary disease. Health and quality of life outcomes. 2008;6(1):46.

37 British Thoracic Society. A Guide to Performing Quality Assured Diagnostic Spirometry 2013 [Available from: https://www.brit-thoracic.org.uk/media/70454/spirometry_e-guide_2013.pdf.

38 Weiss RS. Learning from strangers: The art and method of qualitative interview studies: Simon and Schuster; 1995.

39 Bryman A. Social research methods. Oxford, UK: Oxford university press; 2016.

40 Curtis L, Burns A. Unit Costs of Health and Social Care 2018 Personal Social Services Research Unit (PSSRU): University of kent, Canterbury; 2018 [Available from: http://doi.org/10.22024/UniKent/01.02.70995.

For peer review only

41 NHS DIGITAL. 2017-18 Reference Costs Collection 2018 [Available from: https://digital.nhs.uk/data-and-information/data-collections-and-data-sets/data-collections/reference-costs#2017-18-reference-costs-collection-.

42 O'Brien B, Briggs A. An analysis of uncertainty in health care cost-effectiveness studies: an introduction to statisitical issues and methids. Statistical methods in medical research. 2002;11(6):455-68.

43 Meckley LM, Greenberg D, Cohen JT, Neumann PJ. The adoption of cost-effectiveness acceptability curves in cost-utility analyses. Med Decis Making. 2010;30(3):314-9.

44 Pope C, Ziebland S, Mays N. Qualitative research in health care: analysing qualitative data. BMJ: British Medical Journal. 2000;320(7227):114.

45 INVOLVE. Briefing notes for researchers: involving the public in NHS, public health and social care research. INVOLVE Eastleigh, UK.: NIHR; 2021 [Available from: https://www.nihr.ac.uk/documents/briefing-notes-for-researchers-public-involvement-in-nhs-health-and-social-care-research/27371

46 Barwick M. Developing the Knowledge Translation Plan to Build Research Impact Center on Knowledge Translation for Disability and Rehabilitation Research (KTDRR) Austin, Tx; 2013 [Available from: https://ktdrr.org/training/webcasts/webcast5/webcast_ktplan_101013r.pdf.

For peer review only

Figure 1. Study flow chart

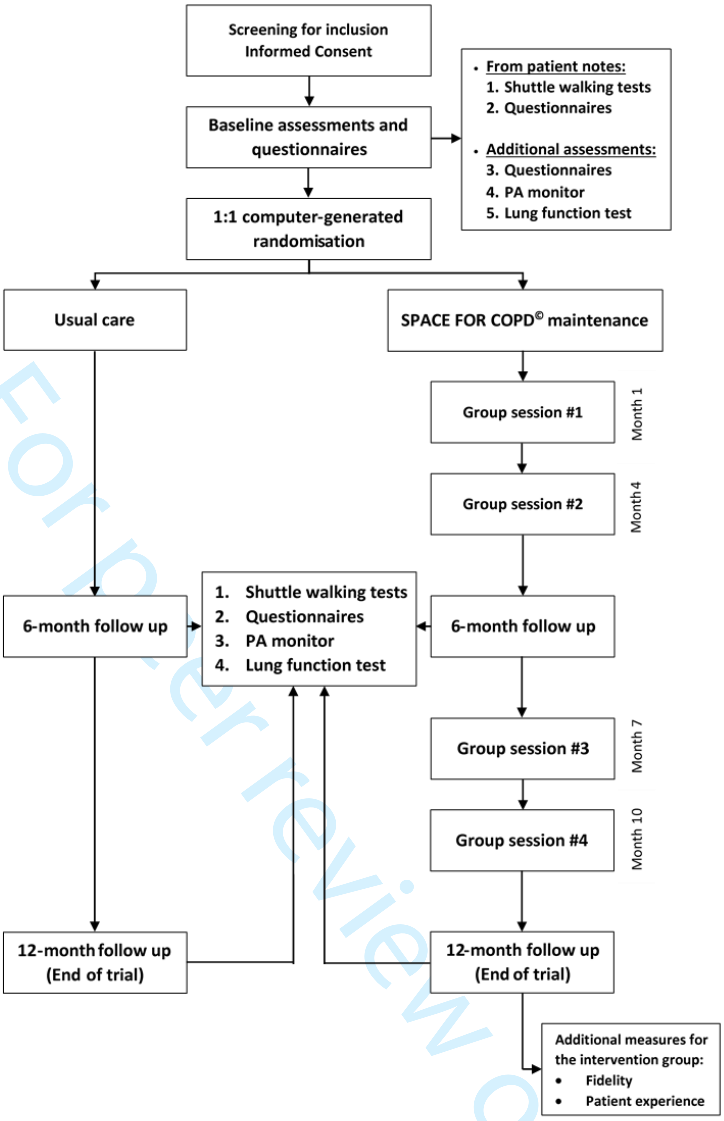

Questionnaires include: Chronic Respiratory Questionnaire-self reported, Chronic Obstructive Pulmonary Disease Assessment Test, EuroQol- five dimensions- five levels, Hospital Anxiety and Depression Scale, and Resource Use Questionnaire.

For peer review only

Figure 2. Group sessions contents

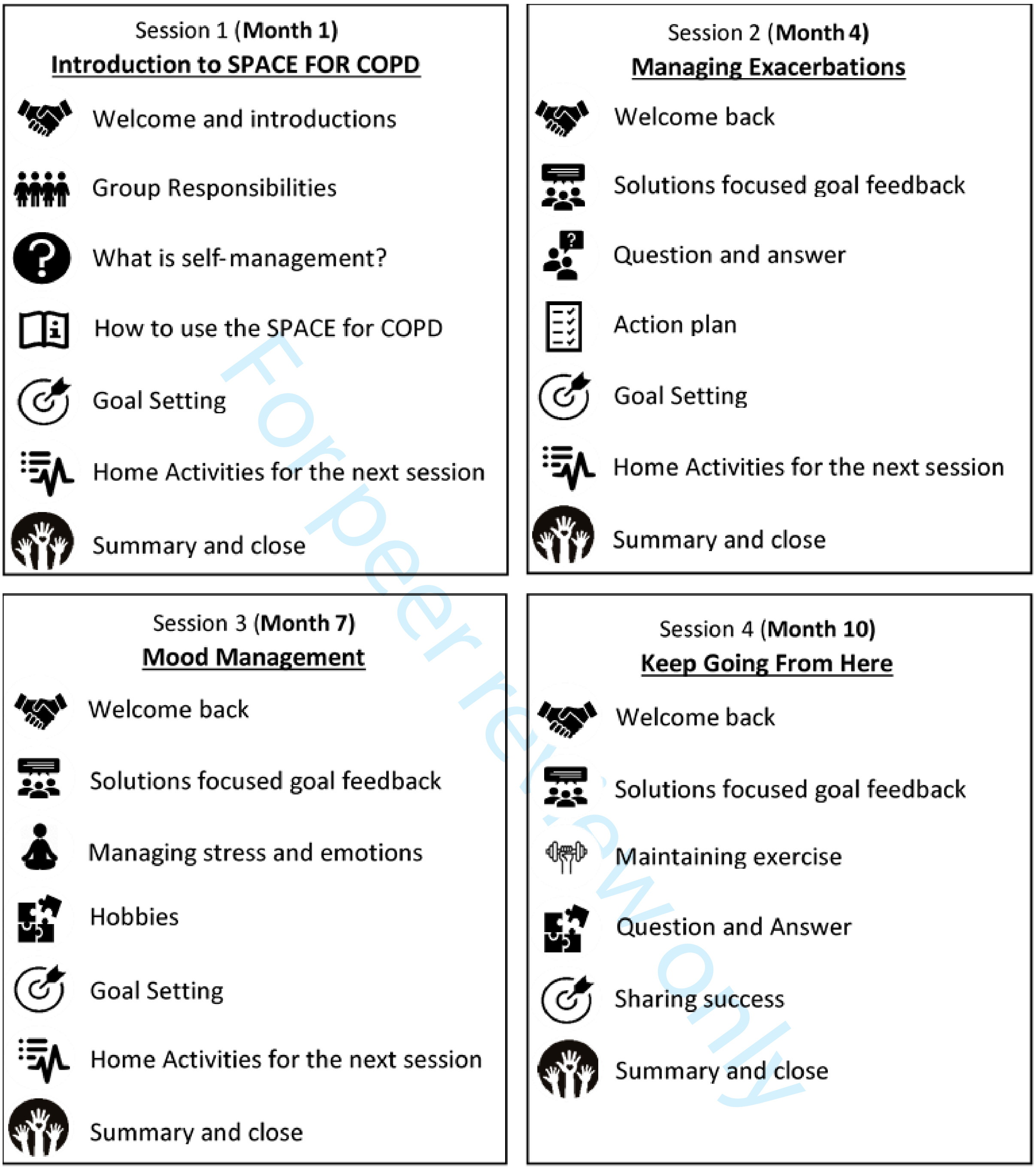

SPIRIT

STANDARD PROTOCOL ITEMS: RECOMMENDATIONS FOR INTERVENTIONAL TRIALS

SPIRIT 2013 Checklist: Recommended items to address in a clinical trial protocol and related documents*

Section/item	Item No	Description	Addressed on page number
**Administrative information**			
Title	1	Descriptive title identifying the study design, population, interventions, and, if applicable, trial acronym	..........1..........
Trial registration	2a	Trial identifier and registry name. If not yet registered, name of intended registry	..........2..........
	2b	All items from the World Health Organization Trial Registration Data Set	..........NA..........
Protocol version	3	Date and version identifier	..........16..........
Funding	4	Sources and types of financial, material, and other support	..........18..........
Roles and responsibilities	5a	Names, affiliations, and roles of protocol contributors	..........18..........
	5b	Name and contact information for the trial sponsor	..........16..........
	5c	Role of study sponsor and funders, if any, in study design; collection, management, analysis, and interpretation of data; writing of the report; and the decision to submit the report for publication, including whether they will have ultimate authority over any of these activities	..........18...............
	5d	Composition, roles, and responsibilities of the coordinating centre, steering committee, endpoint adjudication committee, data management team, and other individuals or groups overseeing the trial, if applicable (see Item 21a for data monitoring committee)	......NA.............

For peer review only

**Introduction**			
Background and rationale	6a	Description of research question and justification for undertaking the trial, including summary of relevant studies (published and unpublished) examining benefits and harms for each intervention	..........2-3..........
	6b	Explanation for choice of comparators	..........3..........
Objectives	7	Specific objectives or hypotheses	..........3-4..........
Trial design	8	Description of trial design including type of trial (eg, parallel group, crossover, factorial, single group), allocation ratio, and framework (eg, superiority, equivalence, noninferiority, exploratory)	..........3..........
**Methods: Participants, interventions, and outcomes**			
Study setting	9	Description of study settings (eg, community clinic, academic hospital) and list of countries where data will be collected. Reference to where list of study sites can be obtained	..........5..........
Eligibility criteria	10	Inclusion and exclusion criteria for participants. If applicable, eligibility criteria for study centres and individuals who will perform the interventions (eg, surgeons, psychotherapists)	..........5..........
Interventions	11a	Interventions for each group with sufficient detail to allow replication, including how and when they will be administered	..........6-7..........
	11b	Criteria for discontinuing or modifying allocated interventions for a given trial participant (eg, drug dose change in response to harms, participant request, or improving/worsening disease)	..........N/A..........
	11c	Strategies to improve adherence to intervention protocols, and any procedures for monitoring adherence (eg, drug tablet return, laboratory tests)	..........7..........
	11d	Relevant concomitant care and interventions that are permitted or prohibited during the trial	..........N/A..........
Outcomes	12	Primary, secondary, and other outcomes, including the specific measurement variable (eg, systolic blood pressure), analysis metric (eg, change from baseline, final value, time to event), method of aggregation (eg, median, proportion), and time point for each outcome. Explanation of the clinical relevance of chosen efficacy and harm outcomes is strongly recommended	..........9-13..........
Participant timeline	13	Time schedule of enrolment, interventions (including any run-ins and washouts), assessments, and visits for participants. A schematic diagram is highly recommended (see Figure)	......5, Figure 1......

For peer review only - http://bmjopen.bmj.com/site/about/guidelines.xhtml

Sample size	14	Estimated number of participants needed to achieve study objectives and how it was determined, including clinical and statistical assumptions supporting any sample size calculations	..........13..........
Recruitment	15	Strategies for achieving adequate participant enrolment to reach target sample size	..........14..........
**Methods: Assignment of interventions (for controlled trials)**			
Allocation:			
Sequence generation	16a	Method of generating the allocation sequence (eg, computer-generated random numbers), and list of any factors for stratification. To reduce predictability of a random sequence, details of any planned restriction (eg, blocking) should be provided in a separate document that is unavailable to those who enrol participants or assign interventions	..........14..........
Allocation concealment mechanism	16b	Mechanism of implementing the allocation sequence (eg, central telephone; sequentially numbered, opaque, sealed envelopes), describing any steps to conceal the sequence until interventions are assigned	..........14..........
Implementation	16c	Who will generate the allocation sequence, who will enrol participants, and who will assign participants to interventions	..........14..........
Blinding (masking)	17a	Who will be blinded after assignment to interventions (eg, trial participants, care providers, outcome assessors, data analysts), and how	..........14..........
	17b	If blinded, circumstances under which unblinding is permissible, and procedure for revealing a participant's allocated intervention during the trial	..........14..........
**Methods: Data collection, management, and analysis**			
Data collection methods	18a	Plans for assessment and collection of outcome, baseline, and other trial data, including any related processes to promote data quality (eg, duplicate measurements, training of assessors) and a description of study instruments (eg, questionnaires, laboratory tests) along with their reliability and validity, if known. Reference to where data collection forms can be found, if not in the protocol	..........9-13 ....
	18b	Plans to promote participant retention and complete follow-up, including list of any outcome data to be collected for participants who discontinue or deviate from intervention protocols	..........11..........

For peer review only - http://bmjopen.bmj.com/site/about/guidelines.xhtml

Data management	19	Plans for data entry, coding, security, and storage, including any related processes to promote data quality (eg, double data entry; range checks for data values). Reference to where details of data management procedures can be found, if not in the protocol	..........14..........
Statistical methods	20a	Statistical methods for analysing primary and secondary outcomes. Reference to where other details of the statistical analysis plan can be found, if not in the protocol	..........15..........
	20b	Methods for any additional analyses (eg, subgroup and adjusted analyses)	..........NA..........
	20c	Definition of analysis population relating to protocol non-adherence (eg, as randomised analysis), and any statistical methods to handle missing data (eg, multiple imputation)	..........15..........
**Methods: Monitoring**			
Data monitoring	21a	Composition of data monitoring committee (DMC); summary of its role and reporting structure; statement of whether it is independent from the sponsor and competing interests; and reference to where further details about its charter can be found, if not in the protocol. Alternatively, an explanation of why a DMC is not needed	.....NA conducted by trial steering group...............
	21b	Description of any interim analyses and stopping guidelines, including who will have access to these interim results and make the final decision to terminate the trial	..........15..........
Harms	22	Plans for collecting, assessing, reporting, and managing solicited and spontaneously reported adverse events and other unintended effects of trial interventions or trial conduct	..........16..........
Auditing	23	Frequency and procedures for auditing trial conduct, if any, and whether the process will be independent from investigators and the sponsor	..........9..........
**Ethics and dissemination**			
Research ethics approval	24	Plans for seeking research ethics committee/institutional review board (REC/IRB) approval	..........16..........
Protocol amendments	25	Plans for communicating important protocol modifications (eg, changes to eligibility criteria, outcomes, analyses) to relevant parties (eg, investigators, REC/IRBs, trial participants, trial registries, journals, regulators)	..........16..........

For peer review only - http://bmjopen.bmj.com/site/about/guidelines.xhtml

Consent or assent	26a	Who will obtain informed consent or assent from potential trial participants or authorised surrogates, and how (see Item 32)	....
	26b	Additional consent provisions for collection and use of participant data and biological specimens in ancillary studies, if applicable	..........N/A..........
Confidentiality	27	How personal information about potential and enrolled participants will be collected, shared, and maintained in order to protect confidentiality before, during, and after the trial	..........14..........
Declaration of interests	28	Financial and other competing interests for principal investigators for the overall trial and each study site	........18...........
Access to data	29	Statement of who will have access to the final trial dataset, and disclosure of contractual agreements that limit such access for investigators	..........15..........
Ancillary and post-trial care	30	Provisions, if any, for ancillary and post-trial care, and for compensation to those who suffer harm from trial participation	..........N/A..........
Dissemination policy	31a	Plans for investigators and sponsor to communicate trial results to participants, healthcare professionals, the public, and other relevant groups (eg, via publication, reporting in results databases, or other data sharing arrangements), including any publication restrictions	..........16-17..........
	31b	Authorship eligibility guidelines and any intended use of professional writers	.......N/A............
	31c	Plans, if any, for granting public access to the full protocol, participant-level dataset, and statistical code	........N/A...........
**Appendices**			
Informed consent materials	32	Model consent form and other related documentation given to participants and authorised surrogates	.......included as appendix............
Biological specimens	33	Plans for collection, laboratory evaluation, and storage of biological specimens for genetic or molecular analysis in the current trial and for future use in ancillary studies, if applicable	..........N/A..........

*It is strongly recommended that this checklist be read in conjunction with the SPIRIT 2013 Explanation & Elaboration for important clarification on the items. Amendments to the protocol should be tracked and dated. The SPIRIT checklist is copyrighted by the SPIRIT Group under the Creative Commons "Attribution-NonCommercial-NoDerivs 3.0 Unported" license.

For peer review only - http://bmjopen.bmj.com/site/about/guidelines.xhtml

BMJ Open

SPACE FOR COPD© delivered as a maintenance programme on Pulmonary Rehabilitation discharge: protocol of a randomised controlled trial evaluating the long-term effects on exercise tolerance and mental wellbeing.

Journal:	*BMJ Open*
Manuscript ID	bmjopen-2021-055513.R2
Article Type:	Protocol
Date Submitted by the Author:	21-Feb-2022
Complete List of Authors:	Alqahtani, Khaled A.; Jazan University, Respiratory Therapy Department; University of Leicester, Respiratory Sciences<br>Gerlis, Charlotte; Glenfield Hospital Respiratory Medicine Department, Centre of Exercise and Rehabilitation Science, Leicester Biomedical Research Centre- Respiratory<br>Nolan, Claire; Royal Brompton and Harefield NHS Foundation Trust, Department of Respiratory Medicine<br>Gardiner, Nikki; Glenfield Hospital Respiratory Medicine Department, Centre of Exercise and Rehabilitation Science, Leicester Biomedical Research Centre- Respiratory<br>Szczepura, Ala; Coventry University, Faculty of Health and Life Sciences<br>Man, William; Royal Brompton and Harefield NHS Foundation Trust, Respiratory Medicine; Imperial College London Faculty of Medicine<br>Singh, Sally; Glenfield Hospital Respiratory Medicine Department, Centre for Exercise and Rehabilitation Science, Leicester Biomedical Research Centre-Respiratory; University of Leicester, Respiratory Sciences<br>Houchen-Wolloff, Linzy; Glenfield Hospital Respiratory Medicine Department, Centre of Exercise and Rehabilitation Science, Leicester Biomedical Research Centre- Respiratory; University of Leicester, Respiratory Sciences
**Primary Subject Heading**:	Respiratory medicine
Secondary Subject Heading:	Rehabilitation medicine, Qualitative research, Health economics
Keywords:	Protocols & guidelines < HEALTH SERVICES ADMINISTRATION & MANAGEMENT, QUALITATIVE RESEARCH, REHABILITATION MEDICINE, RESPIRATORY MEDICINE (see Thoracic Medicine), Chronic airways disease < THORACIC MEDICINE

SCHOLARONE™
Manuscripts

For peer review only - http://bmjopen.bmj.com/site/about/guidelines.xhtml

TITLE PAGE

Authors: Khaled Alqahtani[1,2], Charlotte Gerlis[1], Claire Nolan[3], Nikki Gardiner[1], Ala Szczepura[4], William Man[3,5], Sally J Singh[1,2], Linzy Houchen-Wolloff[1,2].

Corresponding author details: Linzy Houchen-Wolloff PhD. Address: Glenfield Hospital Respiratory Medicine Department, Centre of Exercise and Rehabilitation Science, Leicester Biomedical Research Centre- Respiratory, Leicester, UK. Telephone: 0044 0116 250 2759. Email: Linzy.Houchen@uhl-tr.nhs.uk Fax: n/a.

Co-author details:

1 Glenfield Hospital Respiratory Medicine Department, Centre of Exercise and Rehabilitation Science, Leicester Biomedical Research Centre- Respiratory, Leicester, UK.

2 University of Leicester, Respiratory Sciences, Leicester, UK.

3 Royal Brompton and Harefield NHS Foundation Trust, Respiratory Medicine, London, UK.

4 Coventry University, Faculty of Health and Life Sciences, Coventry, West Midlands, UK.

5 Imperial College London Faculty of Medicine, London, UK.

Key words: Protocols & guidelines, Rehabilitation medicine, Respiratory medicine, Chronic airways disease, Qualitative research.

Word count: 4854

For peer review only

TITLE

SPACE FOR COPD© delivered as a maintenance programme on Pulmonary Rehabilitation discharge: protocol of a randomised controlled trial evaluating the long-term effects on exercise tolerance and mental wellbeing.

ABSTRACT

Introduction The benefits achieved during pulmonary rehabilitation (PR) are known to be sustained for 6-12 months after the initial programme. Several maintenance trials have been conducted but were heterogeneous in terms of duration, frequency and labour cost. There is no consensus on one best strategy. SPACE FOR COPD© (Self-management Programme of Activity, Coping, and Education) is a home-based self-management programme which has been shown previously to be effective in primary and secondary care settings and is to be tested here as a maintenance programme. The aim is to evaluate the efficacy of the SPACE FOR COPD© programme (manual and group sessions), on exercise tolerance and mental wellbeing, compared to usual care following PR in patients with COPD.

Methods and analysis A prospective, multicentre, single-blinded randomised controlled trial requiring 116 participants with a clinical diagnosis of COPD who have finished PR within four weeks will be randomised 1:1 to either a usual care group or a SPACE FOR COPD© programme group. The intervention comprises a home-based manual and four, two-hour group sessions adopting motivational interviewing techniques over 12-months. The primary outcome is endurance capacity measured by the Endurance Shuttle Walking Test at 12 months. Secondary outcomes are; maximal exercise capacity, health-related quality of life, mood, patient activation, physical activity, lung function, and health care costs. The measures will be taken at baseline, 6- and 12-months. Patient interviews and staff focus groups will be conducted to explore barriers, facilitators, and views about the intervention at the end of the study. A framework analysis will be used for the interpretation of qualitative data.

Ethics and dissemination The trial was granted ethical approval from Health Research Authority and Health and Care Research Wales (HCRW19/EM/0267 on 10 Oct 2019). Results will be made available to all stakeholders through a dissemination event, conferences, and peer-reviewed publications.

Trial registration ISRCTN30110012.

For peer review only

STRENGTHS AND LIMITATIONS OF THIS STUDY

- This is a novel maintenance trial that uses an evidence-based home-based manual integrated with motivational interviewing skills to promote long-term self-management and behavioural change.
- Patients with COPD who are registered in our patient and public involvement (PPI) group were invited to review the protocol and two were involved in every aspect of developing the project.
- This is a multi-centre trial, therefore the results may be more generalizable than a single centre.
- The programme is limited to English speakers only who can read at the level of an eight year-old. This may therefore exclude those who do not read in English or have low literacy.

INTRODUCTION

To effectively manage COPD, current national1 and international2 clinical guidelines recommend the addition of pulmonary rehabilitation (PR) to standard pharmacological therapy. PR is a non-pharmacological intervention known to help manage and improve respiratory symptoms in stable COPD conditions3 and is also recommended following a hospital admission for acute exacerbation of COPD.1 Moreover, PR is beneficial to improve exercise capacity,3 4 breathlessness,3 quality of life (QoL),5 anxiety and depression,6 and reduce health care utilisation.7 However, these benefits may only be sustained for 6 to 12 months after PR. 4 A number of factors may affect the ability to maintain exercise capacity and QoL in the long term including: frequency of supervised maintenance exercise; strategies used to improve adherence to maintenance exercise; facilitators and barriers to long-term exercise training; and initial PR programme itself 8. Repeated exacerbations, progression of the disease and existence of co-morbidities may also have an impact4 Additionally, from a psychological perspective, patients with COPD report the feeling of abandonment after the graduation from PR as they lose the support from health care professionals and peers received during PR.9

Many interventions have been introduced to facilitate maintaining the benefits of PR beyond a 6 to 12 months period. Some of those studies revealed that maintenance interventions following PR is of benefit to COPD clients to maintain PR outcomes in the short and long term (6 to 24 months).10-12 However, the interventions were heterogenous in

For peer review only - http://bmjopen.bmj.com/site/about/guidelines.xhtml

terms of duration, setting and level of supervision following initial PR. In addition, patients' motivational and behaviour change strategies play a major role in maintenance but were examined in a limited numbers of the aforementioned maintenance interventions. 10-12

Behaviour change can be supported using motivational interviewing (MI) techniques. MI is used to help coach people to make personal changes in their behaviour.13 MI has been shown to outperform traditional 'advice giving' in the treatment of a broad range of behavioural problems/ diseases.14 To our knowledge, none of the previous maintenance interventions have used this technique per se. In COPD, MI coaching delivered on hospital discharge was shown to have a positive influence on readmissions and quality of life compared to a control group.15

Incorporating an exercise manual with group sessions underpinned by MI techniques has been tested in primary and secondary settings as a home PR alternative compared to usual care16 and traditional (centre-based) PR17. The studies revealed benefits in exercise performance, knowledge, anxiety and fatigue in which some of these outcomes were maintained 6-months after the initial delivery, when compared to usual care16 and non-inferior to PR. 17 More recently the programme has been delivered in groups to enhance peer support in COPD. 18

Thus, for this trial, we propose a supervised maintenance package (SPACE FOR COPD©) with the aim of retaining the expected benefits of PR as well as increasing patients' motivation and to address the feelings of abandonment following traditional PR. We will particularly focus on exacerbation and mood management, as well as promoting regular exercise in our programme. By developing this post PR intervention, we aim to preserve physical and mental well-being, potentially reducing the likelihood of repeat PR attendance and hospital readmissions. In this trial, we hypothesise that patients will have improvements in PR outcomes such as exercise capacity and overall quality of life that will be maintained in the intervention group for at least one year. This also could be cost effective for the health service.

Aims

The overarching aim of the study is to assess whether a self-management maintenance programme (SPACE FOR COPD©), delivered on PR discharge, preserves the anticipated gains made in exercise tolerance and mental well-being at 12-months, compared to usual care alone.

For peer review only - http://bmjopen.bmj.com/site/about/guidelines.xhtml

The primary aim is:

- To compare endurance walking distance at 12-months in those who have completed the SPACE FOR COPD© maintenance programme compared to usual care.

The secondary aims are:

- To compare the following variables at 12-months in patients who have completed the SPACE FOR COPD© maintenance programme versus usual care:
  - Maximum walking distance
  - Quality of life
  - Anxiety and depression
  - Physical activity
  - Symptoms (breathlessness, fatigue, sputum production, cough, sleep, chest tightness)
  - Patient activation (knowledge, skills and confidence to manage COPD)
  - Forced Expiratory Volume in 1 second (FEV_1)
- To measure adherence to the SPACE FOR COPD© maintenance programme in terms of sessions attended, completion of the programme and manual diary entries.
- To collect data for future health economic analysis in both groups; including hospital admissions, health care contacts, personal social service costs, and patient-borne expenditure.
- To understand the patient experience of SPACE FOR COPD©; to identify facilitators and barriers of the package to maintain health and well-being.
- To work with staff, managers and commissioners involved in the delivery of SPACE FOR COPD© as a maintenance option to explore how, if successful, this programme could be rolled-out more widely.
- To use data pre-PR, where available, to evaluate the effectiveness of the initial PR programme and its impact on the maintenance rehabilitation programmes outcomes.

METHODS AND ANALYSIS

Trial design and setting

The trial will be a prospective, multicentre, randomised controlled, single blind trial of SPACE FOR COPD© maintenance programme versus usual care. There will be an integrated economic evaluation and also an embedded qualitative component. The trial will be conducted at two centres, the University Hospitals of Leicester NHS Trust in Leicester,

For peer review only - http://bmjopen.bmj.com/site/about/guidelines.xhtml

UK, and Harefield Pulmonary Rehabilitation Unit, Harefield, UK. We have utilised the SPIRIT-2013 checklist and recommendations as a guide for designing this trial.19

The trial will consist of three visits (baseline, six- and 12-months). Each visit will last for up to two hours where all outcomes will be collected. In addition, a two-hour small group session will be conducted at month 1 after baseline visit then at months 4, 7, and 10 in the intervention group only. See Figure 1, study flow chart. The trial recruitment will take place over one year, with a one year follow up.

Participants

Eligibility criteria for patients

Participants will be included if they meet the following criteria:

- Completed PR within the last four weeks (PR must meet standards defined by the BTS). 20
- Clinical diagnosis of COPD, and
- Able to read and write English to the age of an eight-year-old.
- 18 years and older

Participants will be excluded if they:

- Have a significant disability which limits the daily physical activity, or
- Unable to read and write English due to the nature of the manual's language.

Figure 1

Eligibility criteria for healthcare professionals

Healthcare professionals (HCPs) involved in delivering the intervention and will be invited to participate in focus groups. The inclusion criterion is to deliver one or more sessions. The aim is to explore their experience, the barriers, and facilitators in delivering the group sessions. The HCPs are respiratory physiotherapists, respiratory nurses, or health psychologists with experience in delivering pulmonary rehabilitation. All involved HCPs have received MI training delivered by a health psychologist and introduction on how to deliver the group sessions using a dedicated trial manual.

For peer review only

Procedure

Usual Care (control group)

The control group will receive usual care: written maintenance advice as recommended by the British Thoracic Society (BTS) quality standards.[21] Leaflets will be given to signpost the individual to maintenance options for exercise following PR. A referral to community exercise schemes will be arranged for the control and intervention groups, if available or desired, as this represents best usual care across the UK.

SPACE FOR COPD© maintenance programme (intervention group)

Participants in the intervention group will receive the SPACE FOR COPD© home-based maintenance programme manual and be invited to attend SPACE FOR COPD© group-based maintenance sessions detailed below.

SPACE FOR COPD© home-based manual

Precise details of the rationale, development and efficacy of the work underpinning the SPACE FOR COPD© manual are available elsewhere.[22] The manual is divided into four stages providing an exercise program and covering educational topics. It includes self-management skills, disease specific information and tasks. The content of the manual has been approved by the Plain English Campaign and received the Crystal Mark for Clarity of British English. The content of the manual is outlined in table 1. Table 2 demonstrates the mapping of self-management components of the manual to the PRISMS taxonomy framework. There is a COPD action plan insert which is consistent with the recruiting centres' usual care.

For the purpose of this trial, a new single page insert has been developed to facilitate longer term goal setting by following the SMART (Specific, Measurable, Achievable, Relevant, and Time bound) method for goal settings. HCPs will advise participants on how to use and follow the manual independently at home. Participants will also be asked to complete section of the manual at home (e.g. exercise diaries) and will have the opportunity to call HCPs with questions via telephone.

Group sessions

The group sessions are held in small groups (5 to 10 participants per group) that will accompany the SPACE FOR COPD© maintenance programme manual with the aim of discussing progress, addressing barriers, increasing motivation to maintenance, to enhance health lifestyle behaviours and to encourage social support. The content of these sessions is shown in figure 2. HCPs facilitating the groups will use MI techniques to identify barriers and

For peer review only

facilitators to maintaining exercise, expressing empathy, supporting self-efficacy and 'rolling with resistance'. Group sessions will be facilitated by two HCPs (e.g. Physiotherapists, respiratory specialist nurses, health psychologists). There will be 4 sessions every 3 months over the period of 1 year.

We anticipate that some participants may not be able to attend all the group sessions or may be unable or unwilling to attend the research centre. In this instance, we will accommodate the participant in another session (with permission of that group) or via videoconferencing. If this isn't possible a telephone contact will be conducted instead.

Table 1. Manual components

**Stage 1**<br>What's happened to your lungs?<br>Exercise: How to get fitter<br>Setting your goals<br>Managing your stress - part 1<br>Your emotions<br>Controlling your breathing<br>Information about your medication	**Stage 4**<br>Staying fit and your hobbies<br>Your relationships<br>Dealing with setbacks<br>Sex and your lungs<br>Breathe Easy<br>Conclusion
**Stage 2**<br>How to stay fit<br>Managing days when you feel unwell<br>Saving your energy<br>Diet and feeling unwell<br>Advice for clearing your chest	**FAQs & Appendix**<br>Frequently asked questions<br>Setting your walking speed<br>Help for carers<br>Advice about oxygen<br>Smoking: advice on giving up<br>Cleaning your breathing devices<br>Spare walking diaries<br>Spare strength training diaries
**Stage 3**<br>How to get stronger<br>Managing your stress - part 2<br>Healthy eating<br>Travelling and your lung disease	

For peer review only

Table 2. PRISMS Taxonomy components and examples in the manual

PRISMS Taxonomy Component	Elaboration	Examples from the SPACE FOR COPD© facilitator manual
**Information about condition and/ or its management**	Specific information about COPD is provided within the Stage 1 of the manual.	Participants are provided with information throughout the programme (every session)
**Information about available resources**		Participants are provided with information throughout the programme (every session)
**Provision of/agreement on specific clinical action plans and/or rescue medication**	COPD Action Plan is included as an insert in the manual	Actions plans in session 2
**Practical support with adherence (medication or behavioural)**	Walking and strength training diaries are provided for participants and discussed during sessions	Walking and strength training diaries are provided for participants and discussed during solution focused goal feedback at the beginning of sessions 2, 3 and 4
**Provision of easy access to advice or support when needed**	Participants are able to call programme facilitators between sessions with questions	Participants are provided with contact details for programme facilitators who the can call if needed (throughout programme).
**Training/rehearsal for practical self-management activities**	Including:<br>- Managing exacerbations<br>- Saving your energy	Session 2 covers Managing Exacerbations<br>Session 3 covers Saving your Energy
**Training/rehearsal for psychological strategies**	Including:<br>- Goal setting<br>- Solution focused goal feedback<br>- Problem solving<br>- Self-reward and social reward<br>- Managing stress and emotions	Goal setting activity (including action planning) and solution focused goal feedback (once every session).<br>Problem solving (throughout programme)<br>Managing stress and emotions in session 3.
**Social Support**	Including:<br>- Practical support<br>- Emotional support<br>- Peer support<br>- Group socialising is encouraged	Participants are encouraged to share experiences, advice, ideas and support each other (throughout the programme).
**Lifestyle advice and support**	Including:<br>- Introduction to the walking programme<br>- Strength training	Introduction to walking programme in Session 1<br>Hobbies (Session 3)<br>Maintaining Exercise (Session 4)

For peer review only - http://bmjopen.bmj.com/site/about/guidelines.xhtml

	- Hobbies<br>- Maintaining exercise	

Figure 2

Staff training and intervention fidelity

In total, eight HCPs have been trained to deliver the SPACE FOR COPD© maintenance program by a health psychologist and a specialist nurse. All attended a 1-day training course (supplemented with written material and DVD tutorials to take away) to learn the structure of the structure of the group sessions, the SPACE for COPD manual and MI skills.

The agenda covered: motivational interviewing skills, introduction to the facilitator manual and review of SPACE FOR COPD© manual, practical workshop, group facilitation, learning outcomes re-visited, and trial management. 6 of the HCPs had previously received additional MI training and all had experience in delivering PR.

A tele-conference will be held between all the facilitators across the two sites prior to and following the first intervention session to problem solve and share experiences. The tele-conference will then be continued on a regular basis to maintain the flow of the maintenance procedure and to provide peer support.

Quality Assurance will be undertaken to assess delivery of intervention content and educational style. Intervention fidelity checklists for the intervention facilitators have been adapted from a previous study.18 One session per self-management group will be peer-observed and the checklist will be completed. Each site will observe the opposite site's group. Facilitators will complete fidelity checklists at the end of each session. These will assess: content covered, managing groups and adhering to MI principles.

For the trial, external auditing may take place at the discretion of the sponsor (UHL NHS trust).

Study outcomes

The assessments described below will be conducted at baseline, 6- and 12-month visits and completed by a blinded assessor, unaware of the group allocation. Due to the impact of COVID-19 restrictions, assessment visits and consent may be completed over the phone for all data other than physical tests where this is not possible (i.e. spirometry/ walking tests).

For peer review only

Primary outcome

Endurance capacity

The primary endpoint at 12 months is endurance capacity measured by the Endurance Shuttle Walk Test (ESWT), expressed in seconds. The ESWT is a walking field test that has been found to be safe, valid, and reproducible among patients with COPD.23 The minimal clinically important difference (MCID) for this population is estimated to be between 174 and 279 seconds.24

Secondary outcomes

Exercise capacity

Incremental Shuttle Walking Test (ISWT) will be used to measure the maximal exercise capacity following the test protocol published by Singh, et al.25 The MCID in walking distance is between 35.0 and 36.1 metres.26

Chronic respiratory questionnaire-self reported (CRQ-SR):

CRQ-SR is a valid and reliable self-administered tool to assess health-related quality of life (HRQoL) in people with chronic lung diseases.27 It assesses four domains with MCID defined as a mean change of 0.5 for each domain. The domains are dyspnoea (5 questions), fatigue (4 questions), emotional function (7 questions), and mastery (4 questions) and are scored on a 7-point scale per question.

Chronic obstructive pulmonary disease assessment test (CAT):

CAT is a tool developed to assess and monitor COPD symptoms and changes in patient's health status.28 It is responsive in both short and long term assessments and is used widely in PR programmes for patients with COPD.4 It consists of eight simple questions which cover "cough, phlegm, chest tightness, breathlessness going up hills/stairs, activity limitation at home, confidence leaving home, sleep and energy" on a 5-Likert scale. Scores ranges from 0 to 40 indicating none to very severe impact on health status.

EuroQol- five dimensions – five levels (EQ5D5L):

The EQ5D5L is a self-administered non-disease specific tool used to measure HRQoL and is developed by the EuroQol group.29 This measures health on five dimensions (mobility, self-care, usual activities, pain or discomfort, and anxiety or depression) and a tariff is available for deriving a single utility score based on time trade-off utility scores. It also includes a visual analogue scale on which individuals rank their health today from zero (worst health) to 100 (best health). 30

For peer review only

Patient activation measure:

The NHS long term plan31 supports the engagement of patients to self-management, which is a vital key to our trial, where the 13-item patient activation measure (PAM-13) tool can be used as recommended by NHS.32 PAM-13 tool assesses participants' knowledge, skill and confidence for managing their own health and healthcare.33 It is valid and reliable tool that defines four levels of activations ranges from low level to high level of activation.

Anxiety and depression:

The Hospital Anxiety and Depression Scale (HADS) will be used to measure anxiety and depression.34. It is a responsive tool and it has been tested on patients with COPD in several study settings.35 It has 14 questions to measure anxiety and depression (7 questions for each scale). Minimum and maximum scores for each scale are 0 to 21, respectively. The MCID is a mean change of 1.5 points for each scale.36

Physical activity monitoring:

The participants will be asked to wear a physical activity monitor after each visit (wGT3X- ActiGraph, Pensacola, FL, USA), for 7 consecutive days, except during sleeping and water-based activities. The monitor will be activated automatically for that period and doesn't require the participant to turn it on or off. It is a small device attached to an elastic belt to be worn on the waist and placed at the mid right hip. It will enable us to retrieve daily activity bouts, intensity of activities and step counts. Activity monitors can be posted out if assessment is performed over the phone.

Pulmonary function testing:

Pulmonary function will be assessed by hand-held spirometry called Vitalograph Pneumotrac 6800 (Vitalograph, Buckingham,UK) to confirm and assess the severity of COPD. The test will be performed if there was no previous test done within a three-month period. The spirometry test will be done following standard guidelines.37 The device will be calibrated on daily basis and participants will be assessed for contraindications prior to performing the test.

Patient adherence:

The exercise diary entries embedded within the SPACE FOR COPD© manual will be used to measure adherence to the home-based maintenance programme part. Also, group session attendance will be recorded and calculated as total attendance rate. A 75 per cent

For peer review only

cut off will be considered as a programme completion at the end of the trial. To reduce loss to follow-up we will reimburse participants' travel expenses. Where patients drop-out of the SPACE FOR COPD© maintenance programme, we will still collect 6 and 12-month assessment data if consent is still in place.

Adherence to the programme is critical. To improve adherence we will be as flexible as possible with sessions and closely monitor home exercise diaries. If a participant is unable to attend a session we will offer attendance of the same session within another group (with the verbal consent of that group) or via teleconference as the preferred option or a one to one contact. We will send out text/ email/ postal reminders for group sessions and appointments. We expect that some patients may readmit to hospital and attend PR again. This will be documented but we would not deny further post-exacerbation PR in these patients, in accordance with UK guidelines.[20]

Resource use questionnaires:

Structured resource use questionnaires have been designed for the purpose of this trial and piloted prior to use. These will collect information on the number of hospital inpatient and outpatient episodes; community health & social care costs; and changes to employment status. Patient-borne costs associated with hospital visits (e.g. travel, dependant care, time off work) and out-of-pocket expenditure on products or equipment (e.g. walking shoes) will also be recorded. Questionnaires will be administered to coincide with visit times i.e. baseline, 6- and 12-months.

Interviews with patients:

Semi-structured, face-to-face interviews will be conducted with patients in the intervention group: both completers and non-completers. This will be optional and there will be a separate box on the patients' consent form for participants to opt in (appendix 1). The aim of the interview is to give a more complete picture of the perspective of the subjects of the study[38]; we will be adopting an interpretativist approach.[39] At the end of the 12 month period (or before if a patient decides not to continue the group sessions) we will conduct an interview to explore their experiences of using the new maintenance manual at home, the group sessions and the research process.

Participants will be recruited using a purposeful sampling technique: maximum variation sampling strategy. These interviews (n=10-20 or until data saturation) will be organised and completed by a qualitative researcher who has not been involved in

For peer review only

delivering the intervention. Indicative interview schedules will be used and so may be refined following the first 4-5 interviews. The purpose of this qualitative work is to explore perceptions of SPACE FOR COPD© as a maintenance strategy.

We will explore (Intervention) patients' experiences of SPACE FOR COPD©, identifying barriers and facilitators to the maintenance of well-being facilitated by the programme and to examine whether the sense of abandonment is lessened. The interview schedule will be flexible enough to allow completely unanticipated issues to arise. The interviewer will have adequate experience and expertise to deal with difficult situations (e.g. distress).

The patient interviews will take place at the point of trial completion (at 12-months or earlier if there is attrition). We are interested in the views of patients who do not complete the programme also. Interviews will take place either in hospital or in the patient's home or via phone or video call for those unable to attend the research centre.

Focus group for HCPs:

We will also conduct focus groups with staff involved in delivering the intervention, to understand the relative burden and benefits of this new intervention to their services. Two focus groups with 5-10 participants per group will be organised and conducted by a qualitative researcher who has not been involved in delivering the intervention.

Staff will attend a semi-structured focus group at their workplace and the findings of these will inform the training needs of those providing the intervention. They will also help to ensure that the intervention is moulded in a way that is compatible with existing models of service provision and working practices, which may help to maximise the likelihood of uptake locally and nationally.

Pre PR data

Baseline data from the initial PR will be collected, where available, to evaluate the effectiveness of the PR and its impact on the SPACE FOR COPD© maintenance programme. Data will include: ESWT, ISWT, CRQ-SR, CAT, HADS as these measures are routinely collected as part of PR.

Sample size

The power calculation (for a continuous outcome superiority trial) is based upon the primary outcome measure. We require 116 patients for an 80% chance of finding a 184 second (standard deviation: SD 282 second) difference in the ESWT time between groups at

For peer review only

12-months (at the 5% level). 184 seconds was the between group difference reported in the original primary care SPACE FOR COPD© study16 and is similar to the suggested MCID range for this outcome (174-279 seconds).24 This number includes expected attrition of 20 per cent.

Recruitment and invitation of participants

Participants will be approached and assessed for eligibility on completion of PR by members of the clinical PR services. Interested participants will receive a participant information sheet and given at least 48 hours to consider their participation. After consultation with our patient and public involvement (PPI) group, we propose not to set a threshold for improvement in PR outcomes for patients to be considered for the trial. Those interested will receive an invitation letter to attend a consent research visit.

Randomisation and blinding

Eligible participants will be consented and randomised using a concealed allocation web-based programme (https://www.sealedenvelope.com/). Randomisation will be 1:1 to either usual care (control) or the SPACE FOR COPD© maintenance programme (intervention). Randomisation codes will be blocked per site, providing even numbers of control and intervention patients at each site and a sufficient number of participants for the intervention group sessions to run. Due to the nature of the intervention, the participants and intervention facilitators will not be blinded to group allocation. However, the assessors will be blinded to group allocation. Other blinded assessors are in place in case of blinding exposure by first blinded assessor. Any unintentional unblinding will be documented.

Data collection and management

The REDCAP database housed by University of Leicester will be used to create our paper case report forms (CRFs) and transfer data to the electronic version will be performed periodically. This password protected database has the facility to set limits, flag discrepancies and provides an audit trail of when data has been entered/ amended and by whom. A random check of 10% of the data will be performed by a member of the research team not involved in data entry. Once data is ready for analysis, it can be easily exported to SPSS statistical analysis software. Paper CRFs will not contain patient identifiable data and

For peer review only

will be stored in locked filling cabinets in key coded offices. Data will be collected and stored in accordance with the 2018 UK General Data Protection Regulation (GDPR).

Data analysis

Quantitative analysis

All data will be analysed on an intention-to-treat basis using SPSS software (version 26.0, IBM, UK). We will conduct a separate per-protocol analysis for those who complete the intervention. Continuous variables will be presented as mean (SD), categorical variables as frequencies (percentages). All data will be assessed for outliers, normality and analysed using appropriate parametric and non-parametric statistics. To test the primary outcome, the difference between the 2 groups for the ESWT time will be analysed at 12-months using an independent t-test (or non-parametric equivalent).

Secondary outcome measures will be analysed in the same way. We will also look at changes in all outcomes in individual participants over time (i.e. pre-PR, post-PR, 6-months and 12-months) using a repeated measures ANOVA (or non-parametric alternative). Significance will be set at $p<0.05$ and differences between groups will be expressed as mean (95% confidence intervals). Analysis will take into account the baseline values and adjust for potential confounders (e.g. age, disease severity). Missing data will be corrected for by multiple imputation. Interim data may be made available for abstracts but unmasking will not occur.

Economic analysis

An economic analysis will be performed from a societal perspective (including patient-borne costs) and from a health and social care perspective. Patient use of NHS services will be priced using PSSRU[40] costings or NHS reference costs[41] and include appropriate on-costs. Impact on outcomes (health utility) will be assessed using the EQ-5D questionnaire. A cost-utility analysis will compare incremental costs and marginal benefits, and include 1,000 bootstrapped replication.[42.]

The cost per quality-adjusted life-year (QALY) gained, relative to standard care, will be calculated. Intervention costs will be apportioned fully to the 12-month period. A cost-effectiveness acceptability curve (CEAC) will be generated to indicate the probability that the intervention is cost-effective at various cut-off thresholds for the societal valuation of a QALY.[43] Sensitivity analyses will be undertaken.

For peer review only - http://bmjopen.bmj.com/site/about/guidelines.xhtml

Qualitative analysis

All interviews/ focus groups will be recorded and transcribed verbatim with participants' written consent. Data will be analysed according to the framework analysis approach.44 This approach allows for data collection to start with pre-set objectives whilst ensuring that analysis reflects the experiences of participants. Framework analysis follows 5 stages: familiarisation with data, identification of thematic framework, indexing of data to thematic framework, charting of data to framework and interpretation of the framework.

Harms

Patients will be asked to complete several simple physical tests at the trial visits. They may feel breathless/ tired at the end of the walking tests but this is normal and usually settles within a few minutes. It would be very unusual for symptoms to persist. However, in this unlikely situation, medical staff are on hand at the hospital. We follow a standard operating procedure for walking tests to ensure safety. If any results from the tests undertaken as part of the trial are clinically significant, we will inform both the participant and their GP (with patient consent).

We will abide by the safeguarding policy of the University Hospitals of Leicester NHS Trust and Royal Brompton & Harefield NHS Trust to ensure that all staff working on the trial have undertaken safeguarding training.

Patient and public involvement statements

For this trial we have two PPI members on the project steering committee, who will be involved in all stages of the research cycle. We have one member from each recruiting site to ensure that local issues may be addressed. We know from previous projects how valuable our PPI colleagues can be in terms of helping to shape the research and troubleshooting any issues. For instance, our recent SPACE FOR COPD© pragmatic study (delivering the SPACE FOR COPD© programme to community groups) benefitted from PPI feedback when designing the format and scheduling of the patient group sessions to ensure that the travel arrangements and times were not onerous for patients.

For this project, PPI members have helped us to adapt the protocol in in terms of agreeing the participant inclusion criteria and how the SPACE FOR COPD© maintenance programme should be delivered. The relationship between researchers and PPI members will be collaborative; the reason for this is to ensure that there is an on-going partnership

For peer review only - http://bmjopen.bmj.com/site/about/guidelines.xhtml

between the research team and the service users, where decisions about the research are shared. To help support members we will co-produce all documentation to ensure they are written in lay language. We anticipate that the PPI members will be involved in developing and reviewing patient documents.

We would involve different stakeholders such as other health professionals, commissioners and charities (e.g. the British Lung Foundation Breathe Easy groups) in our discussions. We provide training to our PPI members and have budgeted appropriately to support this level of PPI taken from the INVOLVE guidelines.[45] PPI colleagues will be involved in the dissemination of the work at a local level.

Ethics and dissemination

The trial was granted ethical approval from Health Research Authority and Health and Care Research Wales (HCRW) with Research Ethics Committee number 19/EM/0267 on 10th October 2019. The first participant was recruited on 18/10/2019. The study is sponsored by University Hospitals of Leicester NHS Trust and funded by National Institute for Health Research with grant number PB-PG-0317-20032. Amendments to protocol will go through HCRW and sponsor before enforcement.

In order to have a well-structured dissemination plan, we have adopted the Scientist Knowledge Translation Plan.[46] It guides researchers through the stages of developing a dissemination strategy including clarifying 'key messages' identifying ways to transmit those messages and designing an evaluation plan. See here for details: http://ktdrr.org/training/webcasts/webcast5/webcast_ktplan_101013r.pdf

Audiences for this trial are commissioning organisations (such as CCGs, STPs and NHS England), HCPs providing rehabilitation, patients and the public, external statutory organisations (such as the Department of Health, NICE, CLAHRCs), and Academia. They will be involved throughout the research process to better shape the outcomes and have it easily translated to the community at the end of the trial. The delivery of the trial outcomes will be communicated via face-to-face. Moreover, it will include, but not limited to, development of links with key organisations, use of electronic media, and publications including full and plain English summary reports.

For peer review only - http://bmjopen.bmj.com/site/about/guidelines.xhtml

Author Contribution

KA, CG, CN, NG, AS, WM, SS, LHW- all members of the study steering group.

Contributorship Statement

The authors would like to thank Dr Claire Bourne for her assistance in developing the intervention and staff training. With thanks also to our PPI co-applicants Freda Smart (Leicester) and Chris Hedger (Harefield) and to the clinical rehabilitation teams at both sites for their assistance in refining the protocol.

Funding Statement

This work was funded by the National Institute for Health Research (NIHR) Research for Patient Benefit Programme, grant number [PB-PG-0317-20032] and supported by the NIHR Leicester Biomedical Research Centre (BRC)- Respiratory.

Figure Legend

Figure 1. Study flow chart

Figure 2. Group sessions contents

Competing interests

There are no competing interests for any author.

References

1 National Institute for Health and Clinical Excellence. Chronic obstructive pulmonary disease in over 16s: diagnosis and management 2018 [Available from: https://www.ncbi.nlm.nih.gov/books/NBK542426/.

2 Alison JA, McKeough ZJ, Johnston K, McNamara RJ, Spencer LM, Jenkins SC, et al. Australian and New Zealand Pulmonary Rehabilitation Guidelines. Respirology. 2017;22(4):800-19.

3 Griffiths TL, Burr ML, Campbell IA, Lewis-Jenkins V, Mullins J, Shiels K, et al. Results at 1 year of outpatient multidisciplinary pulmonary rehabilitation: a randomised controlled trial. The Lancet. 2000;355(9201):362-8.

For peer review only

4 Spruit MA, Singh SJ, Garvey C, ZuWallack R, Nici L, Rochester C, et al. An official American Thoracic Society/European Respiratory Society statement: key concepts and advances in pulmonary rehabilitation. Am J Respir Crit Care Med. 2013;188(8):e13-64.

5 McCarthy B, Casey D, Devane D, Murphy K, Murphy E, Lacasse Y. Pulmonary rehabilitation for chronic obstructive pulmonary disease. Cochrane Database Syst Rev. 2015(2):CD003793.

6 Harrison S, Greening N, Williams J, Morgan M, Steiner M, Singh S. Have we underestimated the efficacy of pulmonary rehabilitation in improving mood? Respiratory medicine. 2012;106(6):838-44.

7 Raskin J, Spiegler P, McCusker C, ZuWallack R, Bernstein M, Busby J, et al. The Effect of Pulmonary Rehabilitation on Healthcare Utilization in Chronic Obstructive Pulmonary Disease: THE NORTHEAST PULMONARY REHABILITATION CONSORTIUM. Journal of Cardiopulmonary Rehabilitation and Prevention. 2006;26(4):231-6.

8 Spencer, LM, McKeough, ZJ. Maintaining the benefits following pulmonary rehabilitation: Achievable or not? Respirology. 2019; 24: 909– 915.9 Apps L, Barlow J, Singh S. Attitudes to exercise after a course of pulmonary rehabilitation: the patient's perspective. 2009.

10 Beauchamp MK, Evans R, Janaudis-Ferreira T, Goldstein RS, Brooks D. Systematic review of supervised exercise programs after pulmonary rehabilitation in individuals with COPD. Chest. 2013;144(4):1124-33.

11 Jenkins AR, Gowler H, Curtis F, Holden NS, Bridle C, Jones AW. Efficacy of supervised maintenance exercise following pulmonary rehabilitation on health care use: a systematic review and meta-analysis. Int J COPD. 2018;13:257–73.

12 Malaguti C, Dal Corso S, Janjua S, Holland AE. Supervised maintenance programmes following pulmonary rehabilitation compared to usual care for chronic obstructive pulmonary disease. Cochrane Database of Systematic Reviews 2021, Issue 8. Art. No.: CD013569.

13 Miller WR, Rollnick S. Motivational interviewing: Helping people change: Guilford press; 2012.

14 Rubak S, Sandbaek A, Lauritzen T, Christensen B. Motivational interviewing: a systematic review and meta-analysis. Br J Gen Pract. 2005;55(513):305-12.

15 Benzo R, Vickers K, Novotny PJ, Tucker S, Hoult J, Neuenfeldt P, et al. Health Coaching and Chronic Obstructive Pulmonary Disease Rehospitalization. A Randomized Study. Am J Respir Crit Care Med. 2016;194(6):672-80.

For peer review only - http://bmjopen.bmj.com/site/about/guidelines.xhtml

16 Mitchell KE, Johnson-Warrington V, Apps LD, Bankart J, Sewell L, Williams JE, et al. A self-management programme for COPD: a randomised controlled trial. Eur Respir J. 2014;44(6):1538-47.

17 Horton EJ, Mitchell KE, Johnson-Warrington V, et alComparison of a structured home-based rehabilitation programme with conventional supervised pulmonary rehabilitation: a randomised non-inferiority trialThorax 2018;73:29-36.

18 Bourne CLA, Kanabar P, Mitchell K, Schreder S, Houchen-Wolloff L, Bankart MJG, et al. A Self-Management Programme of Activity Coping and Education - SPACE for COPD(C) - in primary care: The protocol for a pragmatic trial. Bmj Open. 2017;7(7):e014463.

19 Chan AW, Tetzlaff JM, Altman DG, Laupacis A, Gotzsche PC, Krleza-Jeric K, et al. SPIRIT 2013 statement: defining standard protocol items for clinical trials. Ann Intern Med. 2013;158(3):200-7.

20 Bolton CE, Bevan-Smith EF, Blakey JD, Crowe P, Elkin SL, Garrod R, et al. British Thoracic Society guideline on pulmonary rehabilitation in adults. Thorax. 2013;68 Suppl 2(Suppl 2):ii1-30.

21 BTS Pulmonary rehabilitation quality standards. Published by: British Thoracic Society. Publication date: May 2014. Accessible here: https://www.brit-thoracic.org.uk/quality-improvement/quality-standards/pulmonary-rehabilitation/ Last accessed: 15/12/21.

22 Apps LD, Mitchell KE, Harrison SL, Sewell L, Williams JE, Young HM, et al. The development and pilot testing of the self-management programme of activity, coping and education for chronic obstructive pulmonary disease (SPACE for COPD). Int J Chron Obstruct Pulmon Dis. 2013;8:317-27.

23 Revill SM, Morgan MD, Singh SJ, Williams J, Hardman AE. The endurance shuttle walk: a new field test for the assessment of endurance capacity in chronic obstructive pulmonary disease. Thorax. 1999;54(3):213-22.

24 Zatloukal J, Ward S, Houchen-Wolloff L, Harvey-Dunstan T, Singh S. The minimal important difference for the endurance shuttle walk test in individuals with chronic obstructive pulmonary disease following a course of pulmonary rehabilitation. Chronic Respiratory Disease. 2019;16:1479973119853828.

25 Singh SJ, Morgan MD, Scott S, Walters D, Hardman AE. Development of a shuttle walking test of disability in patients with chronic airways obstruction. Thorax. 1992;47(12):1019-24.

26 Evans RA, Singh SJ. Minimum important difference of the incremental shuttle walk test distance in patients with COPD. Thorax. 2019;74(10):994-5.

For peer review only

27 Williams JE, Singh SJ, Sewell L, Guyatt GH, Morgan MD. Development of a self-reported Chronic Respiratory Questionnaire (CRQ-SR). Thorax. 2001;56(12):954-9.

28 Jones PW, Harding G, Berry P, Wiklund I, Chen WH, Kline Leidy N. Development and first validation of the COPD Assessment Test. Eur Respir J. 2009;34(3):648-54.

29 Cazzola M, MacNee W, Martinez F, Rabe KF, Franciosi L, Barnes P, et al. Outcomes for COPD pharmacological trials: from lung function to biomarkers. European Respiratory Journal. 2008;31(2):416-69.

30 EuroQol G. EuroQol--a new facility for the measurement of health-related quality of life. Health policy (Amsterdam, Netherlands). 1990;16(3):199.

31 National Health Services. NHS Long Term Plan 2018 [Available from: https://www.longtermplan.nhs.uk/online-version/chapter-3-further-progress-on-care-quality-and-outcomes/better-care-for-major-health-conditions/respiratory-disease/.

32 National Health Services. New patient activation report launched 2014 [Available from: https://www.england.nhs.uk/2014/05/patient-activation/.

33 Hibbard JH, Mahoney ER, Stockard J, Tusler M. Development and testing of a short form of the patient activation measure. Health services research. 2005;40(6 Pt 1):1918-30.

34 Zigmond AS, Snaith RP. The hospital anxiety and depression scale. Acta Psychiatr Scand. 1983;67(6):361-70.

35 Paz-Díaz H, De Oca MM, López JM, Celli BR. Pulmonary rehabilitation improves depression, anxiety, dyspnea and health status in patients with COPD. American Journal of Physical Medicine & Rehabilitation. 2007;86(1):30-6.

36 Puhan MA, Frey M, Büchi S, Schünemann HJ. The minimal important difference of the hospital anxiety and depression scale in patients with chronic obstructive pulmonary disease. Health and quality of life outcomes. 2008;6(1):46.

37 British Thoracic Society. A Guide to Performing Quality Assured Diagnostic Spirometry 2013 [Available from: https://www.brit-thoracic.org.uk/media/70454/spirometry_e-guide_2013.pdf.

38 Weiss RS. Learning from strangers: The art and method of qualitative interview studies: Simon and Schuster; 1995.

39 Bryman A. Social research methods. Oxford, UK: Oxford university press; 2016.

40 Curtis L, Burns A. Unit Costs of Health and Social Care 2018 Personal Social Services Research Unit (PSSRU): University of kent, Canterbury; 2018 [Available from: http://doi.org/10.22024/UniKent/01.02.70995.

For peer review only

41 NHS DIGITAL. 2017-18 Reference Costs Collection 2018 [Available from: https://digital.nhs.uk/data-and-information/data-collections-and-data-sets/data-collections/reference-costs#2017-18-reference-costs-collection-.

42 O'Brien B, Briggs A. An analysis of uncertainty in health care cost-effectiveness studies: an introduction to statisitical issues and methids. Statistical methods in medical research. 2002;11(6):455-68.

43 Meckley LM, Greenberg D, Cohen JT, Neumann PJ. The adoption of cost-effectiveness acceptability curves in cost-utility analyses. Med Decis Making. 2010;30(3):314-9.

44 Pope C, Ziebland S, Mays N. Qualitative research in health care: analysing qualitative data. BMJ: British Medical Journal. 2000;320(7227):114.

45 INVOLVE. Briefing notes for researchers: involving the public in NHS, public health and social care research. INVOLVE Eastleigh, UK.: NIHR; 2021 [Available from: https://www.nihr.ac.uk/documents/briefing-notes-for-researchers-public-involvement-in-nhs-health-and-social-care-research/27371

46 Barwick M. Developing the Knowledge Translation Plan to Build Research Impact Center on Knowledge Translation for Disability and Rehabilitation Research (KTDRR) Austin, Tx; 2013 [Available from: https://ktdrr.org/training/webcasts/webcast5/webcast_ktplan_101013r.pdf.

For peer review only

Figure 1. Study flow chart

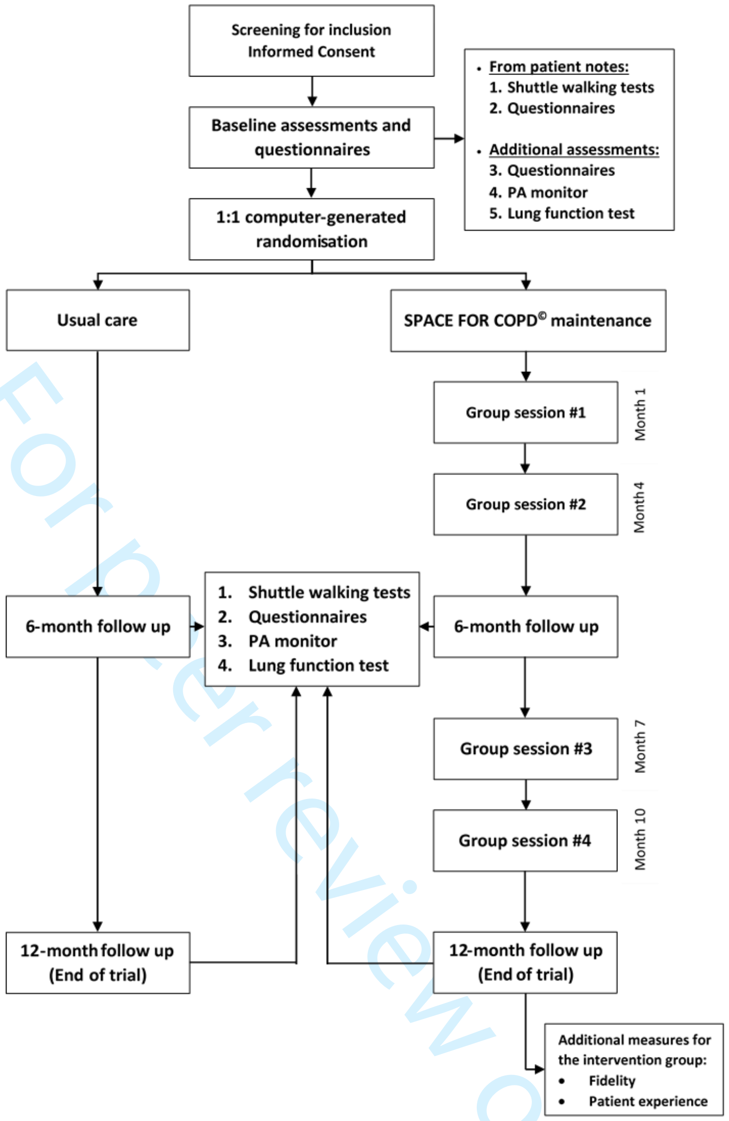

Questionnaires include: Chronic Respiratory Questionnaire-self reported, Chronic Obstructive Pulmonary Disease Assessment Test, EuroQol- five dimensions- five levels, Hospital Anxiety and Depression Scale, and Resource Use Questionnaire.

For peer review only

Figure 2. Group sessions contents

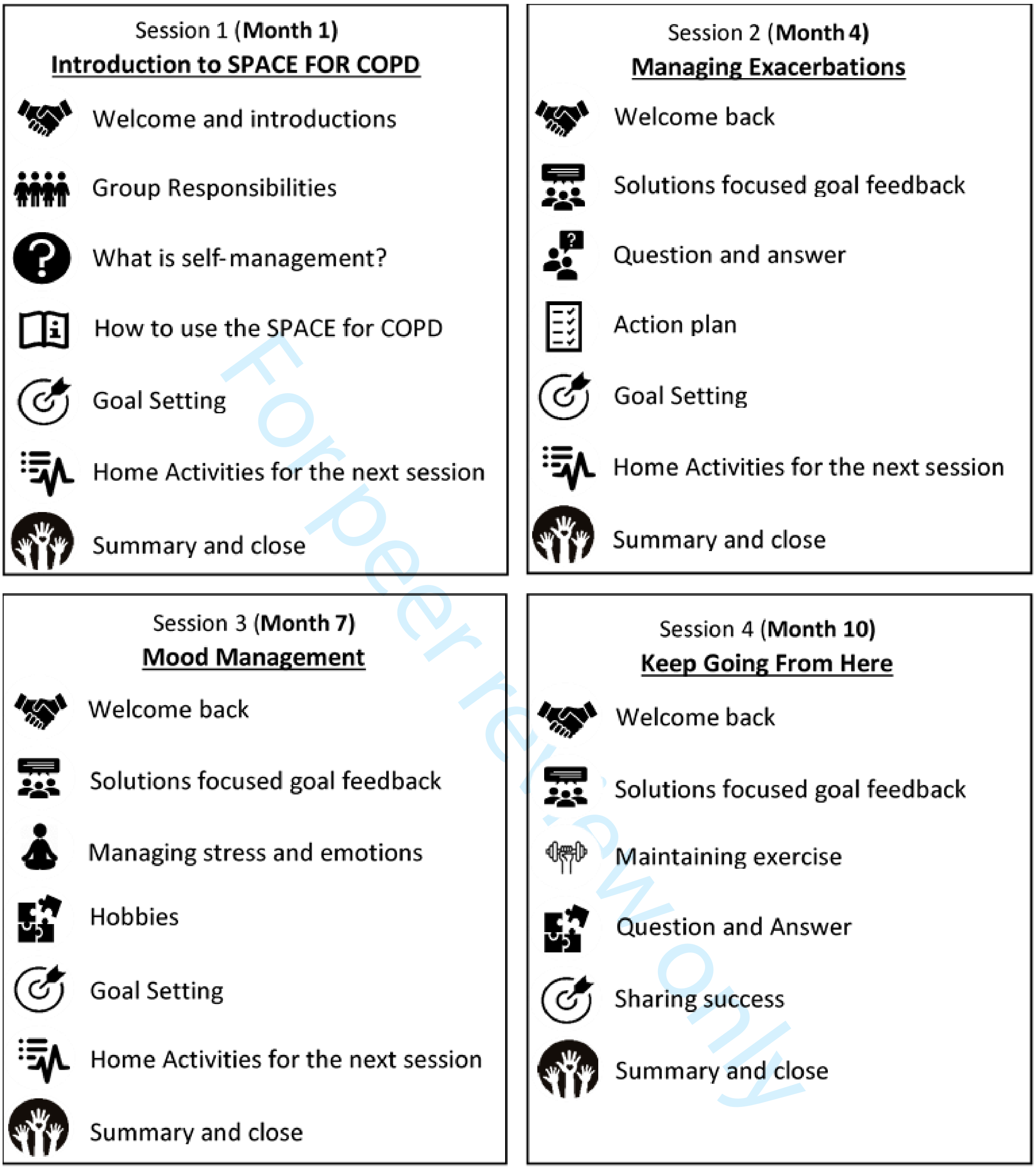

For peer review only

NIHR | Leicester Biomedical Research Centre

Leicester Biomedical Research Centre
Respiratory
Glenfield Hospital
Groby Road
Leicester
LE3 9QP
Tel: 0116 258 3370
Fax: 0116 258 3494
Twitter:@leicesterbrc
Email: NIHRleicesterbrc@uhl-tr.nhs.uk
www.leicesterbrc.nihr.ac.uk

CONSENT FORM FOR PARTICIPANTS

(Version 0.5 on 09/04/2021)

Trial Title: SPACE FOR COPD© delivered as a maintenance programme on Pulmonary Rehabilitation discharge: a randomised controlled trial evaluating the long-term effects on exercise tolerance and mental wellbeing

**Site:**	
**Participant Trial ID number:**	

Please **initial** box

1. I confirm that I have read and understand the information sheet **version 0.5 09/04/2021**, for the above trial, and have had the opportunity to ask questions.	
2. I understand that my participation is voluntary and that I am free to withdraw at any time without giving a reason, and without my medical care or legal rights being affected. I understand that any data collected up until my withdrawal will still be used in the research trial.	
3. I agree to undergo the tests and investigations described in the Participant Information Sheet. The nature of the tests and investigations and any possible risks have been explained.	
4. I understand that relevant sections of my medical notes and data collected during the trial may be looked at by individuals from the trial team, the Sponsor, the funder, regulatory authorities or the NHS Trust, where it is relevant to my taking part in this research. I give permission for these individuals to have access to my records.	
5. I give permission for the clinical data collected during my initial and discharge assessments from my most recent Pulmonary Rehabilitation attendance to be retrieved by the trial team.	
6. I give permission to the trial team to retrieve data about the healthcare services I have utilised over the 12 months of the trial.	
7. I understand that some of my data will be shared with Insignia Health and entered onto a secure database to help improve patient outcome measures, if it is anonymised.	

→→→

For peer review only - http://bmjopen.bmj.com/site/about/guidelines.xhtml

NIHR | Leicester Biomedical Research Centre

Please initial box

8. I understand that my personal details and trial data will be stored on password protected computer systems and paper copies will be kept in a secure office environment.

9. I understand that the information collected about me will be used to support other research in the future, and may be shared anonymously with other researchers.

10. I agree to take part in the above trial. Yes No

11. I agree to take part in the interview at the end of the SPACE maintenance programme which will be audio recorded and transcribed for research purposes. I also agree that anonymised direct quotes can be used for research purposes. Yes No

Name of participant (printed)	Date	Signature

Name of person taking consent (printed)	Date	Signature

Consent form completed: by telephone/ face to face (delete as appropriate)

Original in the TMF Copy to the participant and Copy in medical notes

For peer review only

SPIRIT
STANDARD PROTOCOL ITEMS: RECOMMENDATIONS FOR INTERVENTIONAL TRIALS

SPIRIT 2013 Checklist: Recommended items to address in a clinical trial protocol and related documents*

Section/item	Item No	Description	Addressed on page number
**Administrative information**			
Title	1	Descriptive title identifying the study design, population, interventions, and, if applicable, trial acronym	..........1..........
Trial registration	2a	Trial identifier and registry name. If not yet registered, name of intended registry	..........2..........
	2b	All items from the World Health Organization Trial Registration Data Set	..........NA..........
Protocol version	3	Date and version identifier	..........16..........
Funding	4	Sources and types of financial, material, and other support	..........18..........
Roles and responsibilities	5a	Names, affiliations, and roles of protocol contributors	..........18..........
	5b	Name and contact information for the trial sponsor	..........16..........
	5c	Role of study sponsor and funders, if any, in study design; collection, management, analysis, and interpretation of data; writing of the report; and the decision to submit the report for publication, including whether they will have ultimate authority over any of these activities	..........18...............
	5d	Composition, roles, and responsibilities of the coordinating centre, steering committee, endpoint adjudication committee, data management team, and other individuals or groups overseeing the trial, if applicable (see Item 21a for data monitoring committee)	......NA.............

For peer review only

**Introduction**			
Background and rationale	6a	Description of research question and justification for undertaking the trial, including summary of relevant studies (published and unpublished) examining benefits and harms for each intervention	..........2-3..........
	6b	Explanation for choice of comparators	..........3..........
Objectives	7	Specific objectives or hypotheses	..........3-4..........
Trial design	8	Description of trial design including type of trial (eg, parallel group, crossover, factorial, single group), allocation ratio, and framework (eg, superiority, equivalence, noninferiority, exploratory)	..........3..........
**Methods: Participants, interventions, and outcomes**			
Study setting	9	Description of study settings (eg, community clinic, academic hospital) and list of countries where data will be collected. Reference to where list of study sites can be obtained	..........5..........
Eligibility criteria	10	Inclusion and exclusion criteria for participants. If applicable, eligibility criteria for study centres and individuals who will perform the interventions (eg, surgeons, psychotherapists)	..........5..........
Interventions	11a	Interventions for each group with sufficient detail to allow replication, including how and when they will be administered	..........6-7..........
	11b	Criteria for discontinuing or modifying allocated interventions for a given trial participant (eg, drug dose change in response to harms, participant request, or improving/worsening disease)	..........N/A..........
	11c	Strategies to improve adherence to intervention protocols, and any procedures for monitoring adherence (eg, drug tablet return, laboratory tests)	..........7..........
	11d	Relevant concomitant care and interventions that are permitted or prohibited during the trial	..........N/A..........
Outcomes	12	Primary, secondary, and other outcomes, including the specific measurement variable (eg, systolic blood pressure), analysis metric (eg, change from baseline, final value, time to event), method of aggregation (eg, median, proportion), and time point for each outcome. Explanation of the clinical relevance of chosen efficacy and harm outcomes is strongly recommended	..........9-13..........
Participant timeline	13	Time schedule of enrolment, interventions (including any run-ins and washouts), assessments, and visits for participants. A schematic diagram is highly recommended (see Figure)	......5, Figure 1......

For peer review only - http://bmjopen.bmj.com/site/about/guidelines.xhtml

Sample size	14	Estimated number of participants needed to achieve study objectives and how it was determined, including clinical and statistical assumptions supporting any sample size calculations	..........13..........
Recruitment	15	Strategies for achieving adequate participant enrolment to reach target sample size	..........14..........

Methods: Assignment of interventions (for controlled trials)

Allocation:

Sequence generation	16a	Method of generating the allocation sequence (eg, computer-generated random numbers), and list of any factors for stratification. To reduce predictability of a random sequence, details of any planned restriction (eg, blocking) should be provided in a separate document that is unavailable to those who enrol participants or assign interventions	..........14..........
Allocation concealment mechanism	16b	Mechanism of implementing the allocation sequence (eg, central telephone; sequentially numbered, opaque, sealed envelopes), describing any steps to conceal the sequence until interventions are assigned	..........14..........
Implementation	16c	Who will generate the allocation sequence, who will enrol participants, and who will assign participants to interventions	..........14..........
Blinding (masking)	17a	Who will be blinded after assignment to interventions (eg, trial participants, care providers, outcome assessors, data analysts), and how	..........14..........
	17b	If blinded, circumstances under which unblinding is permissible, and procedure for revealing a participant's allocated intervention during the trial	..........14..........

Methods: Data collection, management, and analysis

Data collection methods	18a	Plans for assessment and collection of outcome, baseline, and other trial data, including any related processes to promote data quality (eg, duplicate measurements, training of assessors) and a description of study instruments (eg, questionnaires, laboratory tests) along with their reliability and validity, if known. Reference to where data collection forms can be found, if not in the protocol	..........9-13 ....
	18b	Plans to promote participant retention and complete follow-up, including list of any outcome data to be collected for participants who discontinue or deviate from intervention protocols	..........11..........

Data management	19	Plans for data entry, coding, security, and storage, including any related processes to promote data quality (eg, double data entry; range checks for data values). Reference to where details of data management procedures can be found, if not in the protocol	..........14..........
Statistical methods	20a	Statistical methods for analysing primary and secondary outcomes. Reference to where other details of the statistical analysis plan can be found, if not in the protocol	..........15..........
	20b	Methods for any additional analyses (eg, subgroup and adjusted analyses)	..........NA..........
	20c	Definition of analysis population relating to protocol non-adherence (eg, as randomised analysis), and any statistical methods to handle missing data (eg, multiple imputation)	..........15..........
**Methods: Monitoring**			
Data monitoring	21a	Composition of data monitoring committee (DMC); summary of its role and reporting structure; statement of whether it is independent from the sponsor and competing interests; and reference to where further details about its charter can be found, if not in the protocol. Alternatively, an explanation of why a DMC is not needed	.....NA conducted by trial steering group..............
	21b	Description of any interim analyses and stopping guidelines, including who will have access to these interim results and make the final decision to terminate the trial	..........15..........
Harms	22	Plans for collecting, assessing, reporting, and managing solicited and spontaneously reported adverse events and other unintended effects of trial interventions or trial conduct	..........16..........
Auditing	23	Frequency and procedures for auditing trial conduct, if any, and whether the process will be independent from investigators and the sponsor	..........9..........
**Ethics and dissemination**			
Research ethics approval	24	Plans for seeking research ethics committee/institutional review board (REC/IRB) approval	..........16..........
Protocol amendments	25	Plans for communicating important protocol modifications (eg, changes to eligibility criteria, outcomes, analyses) to relevant parties (eg, investigators, REC/IRBs, trial participants, trial registries, journals, regulators)	..........16..........

For peer review only - http://bmjopen.bmj.com/site/about/guidelines.xhtml

Consent or assent	26a	Who will obtain informed consent or assent from potential trial participants or authorised surrogates, and how (see Item 32)	....
	26b	Additional consent provisions for collection and use of participant data and biological specimens in ancillary studies, if applicable	..........N/A..........
Confidentiality	27	How personal information about potential and enrolled participants will be collected, shared, and maintained in order to protect confidentiality before, during, and after the trial	..........14..........
Declaration of interests	28	Financial and other competing interests for principal investigators for the overall trial and each study site	........18............
Access to data	29	Statement of who will have access to the final trial dataset, and disclosure of contractual agreements that limit such access for investigators	..........15..........
Ancillary and post-trial care	30	Provisions, if any, for ancillary and post-trial care, and for compensation to those who suffer harm from trial participation	..........N/A..........
Dissemination policy	31a	Plans for investigators and sponsor to communicate trial results to participants, healthcare professionals, the public, and other relevant groups (eg, via publication, reporting in results databases, or other data sharing arrangements), including any publication restrictions	..........16-17..........
	31b	Authorship eligibility guidelines and any intended use of professional writers	.......N/A.............
	31c	Plans, if any, for granting public access to the full protocol, participant-level dataset, and statistical code	........N/A............
**Appendices**			
Informed consent materials	32	Model consent form and other related documentation given to participants and authorised surrogates	.......included as appendix and cited on page 13.............
Biological specimens	33	Plans for collection, laboratory evaluation, and storage of biological specimens for genetic or molecular analysis in the current trial and for future use in ancillary studies, if applicable	..........N/A..........

For peer review only - http://bmjopen.bmj.com/site/about/guidelines.xhtml

*It is strongly recommended that this checklist be read in conjunction with the SPIRIT 2013 Explanation & Elaboration for important clarification on the items. Amendments to the protocol should be tracked and dated. The SPIRIT checklist is copyrighted by the SPIRIT Group under the Creative Commons "Attribution-NonCommercial-NoDerivs 3.0 Unported" license.

For peer review only

For peer review only - http://bmjopen.bmj.com/site/about/guidelines.xhtml
